# Mechanisms of resistance to tyrosine kinase inhibitor‐targeted therapy and overcoming strategies

**DOI:** 10.1002/mco2.694

**Published:** 2024-08-24

**Authors:** Xuejin Ou, Ge Gao, Inbar A. Habaz, Yongsheng Wang

**Affiliations:** ^1^ Division of Thoracic Tumor Multimodality Treatment, Cancer Center, West China Hospital Sichuan University Chengdu China; ^2^ Clinical Trial Center, National Medical Products Administration Key Laboratory for Clinical Research and Evaluation of Innovative Drugs, West China Hospital Sichuan University Chengdu China; ^3^ Department of Biochemistry and Biomedical Sciences McMaster University Hamilton Ontario Canada

**Keywords:** cancer, resistance, targeted therapy, tyrosine kinase inhibitors

## Abstract

Tyrosine kinase inhibitor (TKI)‐targeted therapy has revolutionized cancer treatment by selectively blocking specific signaling pathways crucial for tumor growth, offering improved outcomes with fewer side effects compared with conventional chemotherapy. However, despite their initial effectiveness, resistance to TKIs remains a significant challenge in clinical practice. Understanding the mechanisms underlying TKI resistance is paramount for improving patient outcomes and developing more effective treatment strategies. In this review, we explored various mechanisms contributing to TKI resistance, including on‐target mechanisms and off‐target mechanisms, as well as changes in the tumor histology and tumor microenvironment (intrinsic mechanisms). Additionally, we summarized current therapeutic approaches aiming at circumventing TKI resistance, including the development of next‐generation TKIs and combination therapies. We also discussed emerging strategies such as the use of dual‐targeted antibodies and PROteolysis Targeting Chimeras. Furthermore, we explored future directions in TKI‐targeted therapy, including the methods for detecting and monitoring drug resistance during treatment, identification of novel targets, exploration of dual‐acting kinase inhibitors, application of nanotechnologies in targeted therapy, and so on. Overall, this review provides a comprehensive overview of the challenges and opportunities in TKI‐targeted therapy, aiming to advance our understanding of resistance mechanisms and guide the development of more effective therapeutic approaches in cancer treatment.

## Graphical abstract

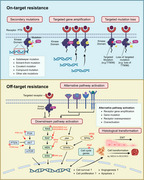

## 1 INTRODUCTION

Protein tyrosine kinases (PTKs) are a group of enzymes that catalyze the phosphorylation of specific tyrosine residues within target proteins.^1^ They play a crucial role in cell signaling pathways, which regulate various cellular processes including cell growth, proliferation, differentiation, and apoptosis.^2^, ^3^ There are two main types of PTKs: receptor PTKs and non‐receptor PTKs.^4^ Receptor PTKs are transmembrane proteins activated by ligand bindings, which leads to autophosphorylation and subsequent signaling cascades. Epidermal growth factor receptor (EGFR), human epidermal growth factor receptor 2 (HER2), platelet‐derived growth factor receptor (PDGFR), fibroblast growth factor receptor (FGFR), insulin receptor, vascular endothelial growth factor receptor (VEGFR), hepatocyte growth factor receptor (also known as mesenchymal epithelial transition, MET), and anaplastic lymphoma kinase (ALK) are classic receptor PTKs, which have been widely and deeply investigated. Non‐receptor PTKs are cytoplasmic or membrane‐associated proteins, which include BCR–ABL fusion protein, Src family kinases, Janus kinases (JAKs), focal adhesion kinase, Syk kinase, and so on.

Aberrant activation of PTKs due to gain‐of‐function mutation, genomic amplification, chromosomal rearrangements, or autocrine activation can lead to dysregulated kinase activities, which ultimately results in uncontrolled cell proliferation and tumorigenesis.^5^, ^6^ A study analyzing pathway alterations in over 9000 tumor samples across 33 cancer types reveals that gene alterations in receptor PTKs and components of the RAS pathway (RTK–RAS pathways) are prevalent in cancer.^7^ The pivotal roles played by PTKs and their widespread abnormal activation in various cancers have made PTKs key targets in cancer therapy. Consequently, tyrosine kinase inhibitors (TKIs) have emerged as prominent anticancer agents in clinical practice, specifically designed to inhibit dysregulated kinases.^8^, ^9^ The first TKI, imatinib (a BCR–ABL TKI), was approved by the United States Food and Drug Administration (US FDA) in May 2001 for the treatment of chronic myeloid leukemia (CML). Since then, there has been significant growth in the development of small‐molecule targeted drugs in malignancies.^8^, ^10^ As of 2021, hundreds of TKIs have been researched, and 76 of them have been approved for clinical use, with most of them for cancer treatment.^8^ Nowadays, TKIs have revolutionized the therapeutic landscape of malignancies, including CML, gastrointestinal stromal tumors (GIST), melanoma, hepatocellular carcinoma (HCC), and non‐small cell lung cancer (NSCLC) (Table 1), ushering in an era of more targeted and effective treatment options.^10^, ^11^, ^12^, ^13^, ^14^ For example, imatinib has transformed CML from a fatal cancer to a chronic disease, by specifically targeting the BCR–ABL fusion protein that drives the proliferation of leukemic cells.^8^ The 8‐year survival of patients with chronic phase CML has significantly improved from ≤15% before 1983 to 87% after the introduction of imatinib in 2001.^15^ In addition, EGFR‐TKIs, such as gefitinib, erlotinib, and osimertinib have become the first‐line treatment for patients with *EGFR*‐mutated NSCLC.^12^, ^16^ EGFR‐TKIs have demonstrated efficacy in prolonging progression‐free survival (PFS) and overall survival (OS) in NSCLC patients with EGFR mutations, sparing patients from low‐response and high‐toxicity platinum‐based chemotherapy.^17^, ^18^

**TABLE 1:** Representative TKIs approved for cancer treatment.

Cancer types	Targets	Alteration prevalence (%)	Treatment	References
CML	*BCR–ABL* gene fusion	100% of CML patients	First‐generation TKIs: imatinib Second‐generation TKIs: dasatinib, nilotinib, bosutinib Third‐generation TKIs: ponatinib (targeting T315I mutation)	^26^
AML	*FLT3* mutations	30% of all AML cases	First‐generation TKIs: sunitinib, sorafenib, midostaurin, lestaurtinib, ponatinib Second‐generation TKIs: quizartinib, gilteritinib, crenolanib	^27^
B‐cell malignancies	Aberrant BTK activation	–	First‐generation TKIs: ibrutinib Second‐generation TKIs: acalabrutinib, zanubrutinib Third‐generation TKIs: pirtobrutinib	^28^
NSCLC	*EGFR* mutations (e.g., ex19del, L858R)	85–90% of EGFR‐mutant NSCLC patients	First‐generation TKIs: gefitinib, erlotinib Second‐generation TKIs: afatinib, dacomitinib Third‐generation TKIs: osimertinib (targeting T790M mutation) Fourth‐generation TKIs: EAI045, JBJ‐04‐125‐02, BLU‐945/BLU‐701	^23^
	*ALK* fusion	3–7% of NSCLC patients	First‐generation TKIs: crizotinib Second‐generation TKIs: alectinib, ceritinib, brigatinib Third‐generation TKIs: Lorlatinib Fourth‐generation TKIs: TPX‐31, NVL‐655	^23^
	*EGFR* mutation (ex20ins)	1.5–2.5% of NSCLC patients	Mobocertinib, amivantamab	^29^
	*ROS1* fusion	1–2%	Crizotinib, entrectinib, ceritinib, ensartinib, brigatinib, lorlatinib, repotrectinib, taletrectinib	^30^
	*RET* fusion	0.7–2% of NSCLC patients	Pralsetinib, selpercatinib	^31^, ^32^
	*BRAF* V600E	1.5–3.5% of NSCLC patients	Dabrafenib+trametinib	^33^
	*KRAS* G12C	20–40% of lung adenocarcinoma patients ∼5% of lung squamous patients	Sotorasib, adagrasib	^34^
	*HER2* alterations	2–3% of NSCLC patients	Trastuzumab deruxtecan	^35^
	*NTRK* 1/2/3 fusion	0.2% of NSCLC patients	First‐generation TKIs: larotrectinib, entrectinib Second‐generation TKIs: selitrectinib, repotrectinib, taletrectinib	^36^
GIST	*KIT* mutations	∼75% of GIST patients	Imatinib, wunitinib	^37^
	*PDGFRA*	5–7% of GIST patients		
Melanoma	*BRAF* mutations	∼50% of cutaneous melanoma patients ∼20% of acral melanoma ∼6% of mucosal melanoma	Vemurafenib, dabrafenib,	^38^
MTC	*RET* mutations	25–40% sporadic MTC	Selpercatinib, pralsetinib	^39^, ^40^
PTC	*RET* fusion	2.5–73% of sporadic PTC	Sunitinib, selpercatinib, pralsetinib	^41^, ^42^, ^43^, ^44^
	*RAS mutations*	10–20% of PTC	Not available	
	*NTRK* fusions	2−13% of PTC	First‐generation TKIs: larotrectinib, entrectinib Second‐generation TKIs: selitrectinib, repotrectinib, taletrectinib	
	*BRAF* fusion	1% of sporadic PTC	Vemurafenib, dabrafenib	
CRC	*KRAS/NRAS* mutations	35–40% of metastatic CRC patients	Not available	^45^
	*BRAF* V600E	5–10% of metastatic CRC patients	Encorafenib+cetuximab	
BRCA	HER2 overexpression	∼15% of breast cancer patients	Pertuzmab, trastuzumab, lapatinib, neratinib, pyrotinib, tucatinib, margetuximab	^46^
HCC	*VEGFR*, *PDGFRβ, RAF1, BRAF, KIT*, etc.	–	Sorafenib, lenvatinib, regorafenib, cabozantinib, ramucirumab	^47^
RCC	*VEGFR1/2/3*, etc.	–	Sunitnib, pazopanib, sorafenib, everolimus, axitnib, cabozantinib, lenvatinib	^48^

This table includes selected cancer types and TKIs and does not aim to represent the entirety of all available TKIs in various cancer types. “–” stands for not applicable.

Abbreviations: ALK, anaplastic lymphoma receptor tyrosine kinase; AML, acute myeloid leukemia; BCR–ABL, breakpoint cluster region‐Abelson murine leukemia; BRAF, B‐Raf proto‐oncogene; BRCA, breast carcinoma; BTK, Bruton tyrosine kinase; CML, chronic myeloid leukemia; CRC, colorectal carcinoma; EGFR, epidermal growth factor receptor; ex19del, exon 19 deletion; ex20ins, exon 20 insertion; FLT3, FMS‐like tyrosine kinase 3; GIST, gastrointestinal stromal tumors; HCC, hepatocellular carcinoma; HER2, human epidermal growth factor receptor 2; KIT, KIT proto‐oncogene; KRAS, kirsten rat sarcoma viral oncogene; MTC, medullary thyroid carcinoma; NSCLC, non‐small cell lung cancer; NTRK, neurotrophic receptor tyrosine kinase; PDGFR, platelet‐derived growth factor receptor; PTC, papillary thyroid carcinoma; RAS, rat sarcoma virus oncogene; RCC, renal cell carcinoma; RET, RET proto‐oncogene; RMS, rhabdomyosarcoma; ROS1, ROS proto‐oncogene 1; SCLL, stem cell leukemia/lymphoma syndrome; TKI, tyrosine kinase inhibitor; VEGFR, vascular endothelial growth factor receptor.

Despite the great success of TKIs, resistance can develop over time. Resistance to TKIs has remained an ongoing and fundamental challenge in cancer‐targeted therapy. Approximately 10−40% of patients receiving front‐line imatinib treatment eventually necessitate an alternative treatment due to intolerance or resistance.^19^, ^20^ Resistance to EGFR‐TKIs is also inevitable in advanced NSCLC patients. Virtually all patients developed acquired resistance within 1−2 years. The median PFS of patients receiving any generation (first, second, or third) of EGFR‐TKIs as first‐line treatment in phase III clinical trials ranges from 8 to 13.7 months.^21^, ^22^ Similar situations can be observed in advanced *ALK*‐positive NSCLC patients receiving crizotinib treatment. Acquired resistance commonly occurs to patients within 1 year with a median PFS of 7.7–10.9 months.^23^ Furthermore, the majority of patients treated with TKIs do not achieve complete remission, with most experiencing only partial remission, indicating that targeted therapies cannot completely eliminate tumor cells, as some tumor cells exhibit intrinsic resistance to TKIs. This phenomenon is also observed in patients undergoing neoadjuvant‐targeted therapy. The pathological complete response rate to neoadjuvant therapy of EGFR‐TKIs was shown to be low (0–12%), and neither was the major pathological response rate high (8–24%).^24^, ^25^

Various mechanisms of resistance to TKIs, including both primary and acquired resistance, have been investigated. Primary resistance to TKIs typically arises from concurrent genetic alterations. The mechanisms of acquired resistance are usually complex and diverse, including both on‐target and off‐target mechanisms. In this review, we summarized the molecular mechanisms of resistance to TKIs, highlighted the overcoming strategies, and discussed the future directions of cancer‐targeted therapy in research and clinical practice.

## 2 MECHANISMS OF RESISTANCE TO TKI‐TARGETED THERAPY

Although TKIs have shown significant efficacy, previous experiences with targeted therapies suggest that resistance to TKIs, whether first‐generation, second‐generation, or third‐generation, is inevitable and remains a critical unresolved challenge. Therefore, further research to elucidate the potential mechanisms of resistance to these drugs is necessary. In the following sections, we focus on discussing the known mechanisms of TKI resistance, broadly categorized as acquired resistance and intrinsic resistance.

### 2.1 Acquired resistance

**FIGURE 1:**
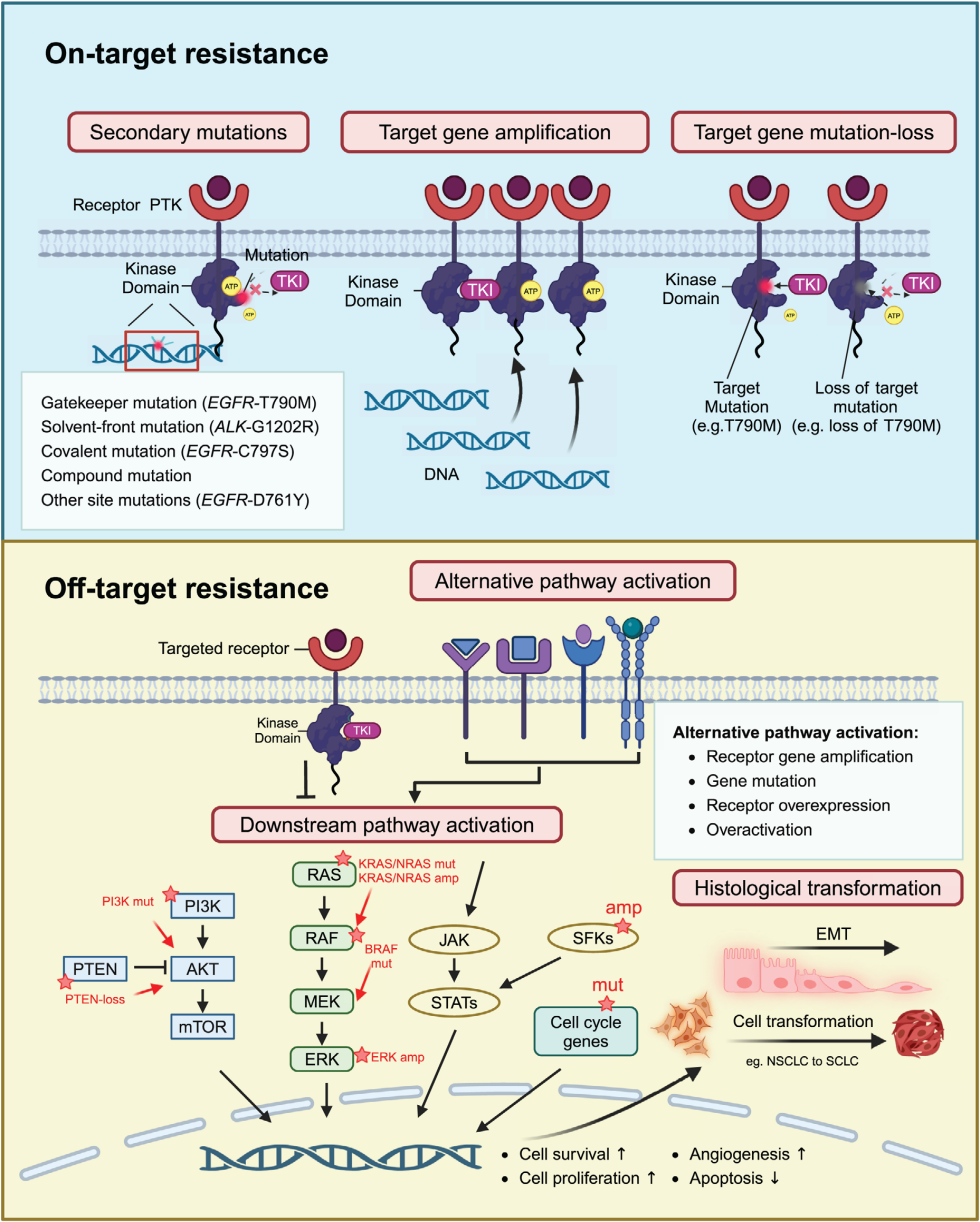
Mechanisms of acquired resistance to TKIs. Mechanisms of acquired resistance to TKIs can be classified as on‐target resistance mechanisms and off‐target resistance mechanisms. Mechanisms of on‐target resistance can be further divided into secondary mutations in targeting kinase domain, targeted gene amplification, and targeted mutation loss. Secondary mutations include gatekeeper mutations, solvent‐front mutations, covalent mutations, compound mutations, and other site mutations. The off‐target mechanisms include alternative pathway activation (parallel receptor activation), downstream pathway activation (mutations or amplification of downstream kinases), and histological transformation (such as EMT and NSCLC to SCLC transformation). ATP, adenosine triphosphate; amp, amplification; EMT, epithelial to mesenchymal transition; NSCLC, non‐small cell lung cancer; mut, mutation; PTK, protein tyrosine kinase; SCLC, small cell lung cancer; TKI, tyrosine kinase inhibitor. This figure is created with BioRender.com.

Acquired resistance to TKIs manifests as resistance that emerges after an initial positive response to TKI treatment. Compared with primary resistance, acquired resistance is more frequently observed in TKI‐targeted therapy. Acquired resistance can arise from various factors including secondary mutations in the targeted tyrosine kinase, overexpression or amplification of the target proteins/genes, loss of the original targeted mutations, activation of alternative signaling pathways, and tumor histological transformation (Figure 1).

#### 2.1.1 Mutations in the target kinase domain (on‐target)

**FIGURE 2:**
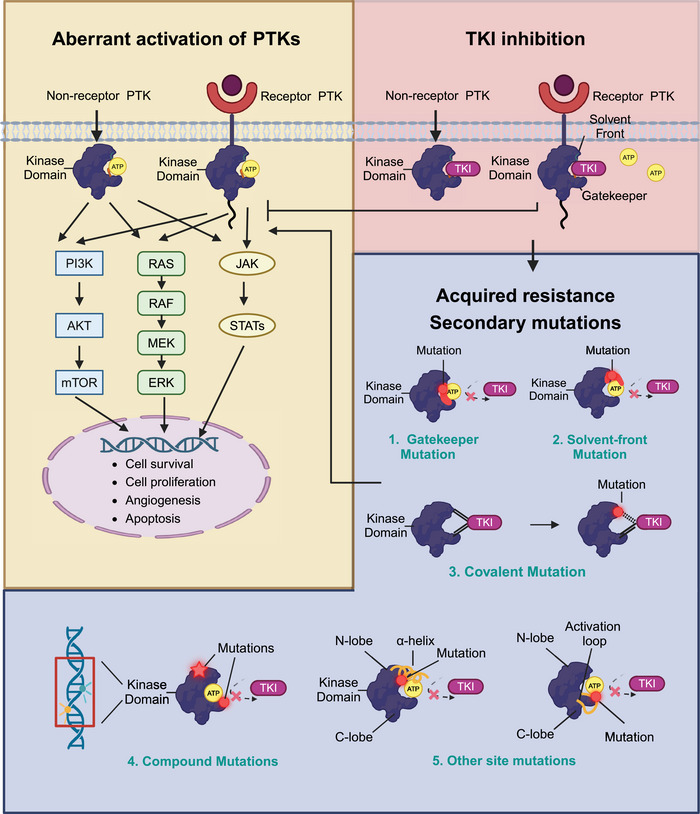
Schematic diagram of secondary mutations conferring TKI resistance. When PTKS are aberrantly activated, the binding of ATP to the tyrosine kinase domain can activate downstream signaling pathways, promoting cancer cell survival, cell proliferation and angiogenesis, and downregulating apoptosis in cancer cells. TKIs can competitively bind to kinase domains and inhibit the activation of downstream signaling pathways, leading to cancer cell apoptosis and cell death. Secondary mutations in the targeting kinase domain represent the most commonly identified acquired resistance mechanisms. Gatekeeper residues situate in the hinge region of tyrosine kinases’ ATP‐binding pocket. Gatekeeper mutation usually involves the substitution of an amino acid with a bulkier amino acid, resulting in a steric hindrance interfering with the binding of TKI to the kinase domain. Solvent‐front mutations refer to amino acid residues located near the solvent‐accessible surface of the ATP‐binding pocket are mutated. Generally, solvent‐front mutations can lead to conformational changes in the target kinase domain, diminish the affinity of TKIs for mutant kinases. Covalent mutations typically happen at the site where TKIs form covalent bonds with tyrosine kinases. These mutations result in the disruption of covalent bonds between TKIs and tyrosine kinases. Compound mutations refer to the presence of two or more mutations within the same gene or across different genes in the same molecule, which have the potential to confer cross‐resistance to multiple TKIs through steric hindrance mechanisms. In addition to the classic secondary mutations discussed above, mutations in other sites of target kinase domains have also been reported, such as the mutations that happen at the αC helix in the N‐terminal lobe and activation loop in the C‐terminal lobe. ATP, adenosine triphosphate; PTK, protein tyrosine kinase; TKI, tyrosine kinase inhibitor. This figure is created with BioRender.com.

Secondary mutations in the target kinase domain, which are also called on‐target mutations, are the most commonly encountered causes of TKI‐acquired resistance. According to the location and function of mutated genes in the target kinase domain, secondary on‐target mutations can be categorized into five types (Figure 2): (1) gatekeeper mutations, (2) solvent‐front mutations, (3) covalent binding site mutations, (4) compound mutations, and (5) other site mutations.

##### Gatekeeper mutation

Among all types of on‐target mutations, gatekeeper mutation is the most frequently identified and extensively investigated TKI‐resistance mechanism. Gatekeeper residues situate in the hinge region of tyrosine kinases’ ATP‐binding pocket.^49^ They play a central role in controlling the accessibility of TKIs to the ATP‐binding pocket.^49^ The mutation of gatekeeper residues can influence the interaction between the inhibitors and their targeting kinases, thereby reduce the efficacy of TKIs and lead to drug resistance.^50^, ^51^ A gatekeeper point mutation, involving the substitution of threonine with isoleucine at position 315 (T315I) in the BCR–ABL protein, was identified in CML patients resistant to imatinib in 2001.^52^ Molecularly, the hydroxyl group of threonine 315 can form a hydrogen bond with imatinib upon binding, while the side chain located at position 315 also regulates the steric interaction, governing the inhibitor's binding to hydrophobic regions neighboring the ATP‐binding pocket.^53^, ^54^ The substitution of threonine with isoleucine leads to the elimination of the hydrogen bond between imatinib and targeted kinase, destabilizing the placement of imatinib.^54^ Therefore, a point mutation of gatekeeper is sufficient to confer imatinib resistance in patients with CML.

Similarly, a gatekeeper mutation in the kinase domain of EGFR (T790M) was found in 50−60% of NSCLC patients resistant to the first‐ and second‐generation EGFR‐TKIs.^55^, ^56^, ^57^ The substitution of threonine with methionine in the catalytic cleft of EGFR kinase domain, *EGFR* T790M, was first identified in a NSCLC patient with disease progression after gefitinib treatment in 2005.^57^ The methionine substitution introduced a bulkier amino acid side chain to the ATP‐kinase‐binding pocket, which resulted in a steric hindrance interfering with the binding of gefitinib or erlotinib.^57^ In addition, the presence of T790M mutation was also proven to enhance the affinity of ATP to the EGFR kinase domain.^58^ As EGFR‐TKIs, such as gefitinib, compete with ATP for binding to the kinase active site, the increased ATP affinity reduces the potency of the inhibitor.^58^ Later on, other gatekeeper mutations (Table 2), such as *ALK*‐L1196M, *ROS1*‐L2026M, *FGFR1*‐V561F/M, *FGFR2*‐V564F/I, *FGFR3‐*V555E, *FGFR4*‐V550L/E/M, *RET*‐V804L/M, *FLT3*‐F691I/L, *KIT* ‐T670I, and *PDGFRα*‐T674I, were identified in patients with IGST, NSCLC, or other cancers after corresponding TKI treatment.^59^

**TABLE 2:** Representative and commonly reported gatekeeper mutations leading to TKI resistance.

PTKs	Mutations	Cancer types	Prior TKIs	Prevalence (%)	References
BCR–ABL	T315I	CML	Imatinib	7%	^52^, ^60^, ^61^
		ALL	Dasatinib	70%	^62^
EGFR	T790M	NSCLC	First‐ or second‐generation EGFR‐TKIs	50–60%	^23^, ^57^
ALK	L1196M/Q	NSCLC	Crizotinib	7%	^63^
			Alectinib	6%	
		ALCL	Alectinib	Cases reported *	^64^
ROS1	L2026M	NSCLC	Crizotinib	8%	^65^
RET	V804L/M	NSCLC	Vandetanib	Cases reported *	^66^, ^67^, ^68^
		MTC	Multikinase inhibitors	Cases reported *	
KIT	T670I	GIST	Imatinib	Cases reported *	^69^
PDGFRα	T674I	GIST	Imatinib	Cases reported *	^70^, ^71^
BTK	T474I	CLL	Pirtobrutinib	22%	^72^
FGFR1	V561M	SCLL	AZD4547, E3810 (lucitanib)	Cases reported *	^73^, ^74^
FGFR2	V564F	CCA	BGJ398	Cases reported *	^75^
FGFR3	V355M/L	Myeloma	AZ12908010	Cases reported *	^74^, ^76^
FGFR4	V550L/M	HCC, RMS	BLU‐554	Cases reported *	^77^, ^78^
NTRK1	F589L	CCA, CRC	Larotrectinib	Cases reported *	^79^, ^80^
FLT3	F691I/L	AML	Quizartinib, gilteritinib	Cases reported *	^81^

This table includes selected gatekeeper mutations and does not aim to represent the entirety of all reported gatekeeper mutations.

“Cases reported *” indicates cases with corresponding mutations were reported in case reports, with no available prevalence rate.

Abbreviations: ALCL, anaplastic large cell lymphoma; ALK, anaplastic lymphoma receptor tyrosine kinase; ALL, acute lymphoblastic leukemia; AML, acute myeloid leukemia; BCR–ABL, breakpoint cluster region‐Abelson; BTK, Bruton tyrosine kinase; CCA, cholangiocarcinoma; CLL, chronic lymphocytic leukemia; CML, chronic myeloid leukemia; CRC, colorectal carcinoma; EGFR, epidermal growth factor receptor; FGFR, fibroblast growth factor receptor; FLT3, FMS‐like tyrosine kinase 3; GIST, gastrointestinal stromal tumors; HCC, hepatocellular carcinoma; KIT, KIT proto‐oncogene; MTC, medullary thyroid carcinoma; NSCLC, non‐small cell lung cancer; NTRK, neurotrophic receptor tyrosine kinase; PDGFR, platelet‐derived growth factor receptor; PTKs, protein tyrosine kinases; RET, RET proto‐oncogene; RMS, rhabdomyosarcoma; ROS1, ROS proto‐oncogene 1; SCLL, stem cell leukemia/lymphoma syndrome; TKI, tyrosine kinase inhibitor.

##### Solvent‐front mutation

Another predominant on‐target mutation mechanism leading to TKI‐resistance is solvent‐front mutation, which confers acquired resistance to *ALK, ROS1, RET, NTK1, NTK2*, and *NTK3* fusion‐targeted therapy.^63^, ^82^, ^83^, ^84^, ^85^, ^86^ Solvent‐front mutations occur in amino acid residues that are located near the solvent‐accessible surface of the ATP‐binding pocket.^87^ For example, solvent‐front mutations *ALK*‐G1202R and *ALK*‐S1206Y which sit near the crizotinib‐binding site are reported to cause acquired resistance to crizotinib in NSCLC patients.^63^, ^87^ Computational modeling suggests that the G1202R mutation results in a bulkier basic residue that would induce steric hindrance, potentially impeding the binding of crizotinib to targeted kinases. And the S1206Y mutation may disrupt the stability of the interaction between the side‐chain hydroxyl group of Ser^1206^ and the carboxylate group of D^1203^.^87^ Generally, solvent‐front mutations lead to conformational changes in the target kinase domain, diminishing the affinity of crizotinib for the mutant *ALK*, and thus abrogating the efficacy of crizotinib to inhibit aberrantly activated *ALK*.^87^ *ROS1*‐G2032R, involving the substitution of glycine with arginine in the solvent front of ROS1 kinase domain, was found to mediate resistance to crizotinib and lorlatinib in *ROS1* fusion‐positive NSCLC patients.^83^, ^88^ Analogous solvent‐front mutations have also been identified in other tyrosine kinases and confer to TKI‐resistance, such as *RET*‐G810 solvent mutations after selpercatinib treatment in NSCLC patients,^84^, ^85^ *EGFR*‐G706S/R mutation following osimertinib treatment in NSCLC patients,^89^ *TRKA*‐G595R and *TRKA*‐G667C mutations after entrectinib treatment in colorectal cancer patients.^86^ The most frequently reported solvent‐front mutations are summarized in Table 3.

**TABLE 3:** Representative and commonly reported solvent‐front mutations leading to TKI resistance.

PTKs	Mutations	Cancer types	Prior TKIs	Prevalence (%)	References
EGFR	G796S/R L792X	NSCLC	Osimertinib	Cases reported *	^89^, ^90^
ALK	G1202R	NSCLC	Crizotinib	2%	^63^
			Alectinib	21–29%	
			Ceritinib		
			Brigatinib		
	I1171T	NSCLC	Crizotinib	2%	^63^
			Alectinib	12%	
RET	G810R/S/C	NSCLC	Selpercatinib	Cases reported *	^85^
ROS1	G2032R	NSCLC	Crizotinib	38%	^88^
			Lorlatinib	32%	
	D2033N	NSCLC	Crizotinib	2.4%	^88^
	L2026M	NSCLC	Crizotinib	Cases reported *	^88^
TRKA	G595R	CRC	Entrectinib	Cases reported *	^79^
			Larotrectinib	Cases reported *	
TRKC	G623R	MASC	Entrectinib	Cases reported *	^79^
			Larotrectinib	Cases reported *	
BTK	C481S	CLL	Ibrutinib	50−75%	^91^, ^92^

This table includes selected solvent‐front mutations and does not aim to represent the entirety of all reported solvent‐front mutations.

“Cases reported *” indicates cases with corresponding mutations were reported in case reports, with no available prevalence rate.

Abbreviations: ALK, anaplastic lymphoma receptor tyrosine kinase; BTK, Bruton tyrosine kinase; CLL, chronic lymphocytic leukemia; CRC, colorectal carcinoma; EGFR, epidermal growth factor receptor; NSCLC, non‐small cell lung cancer; PTKs, protein tyrosine kinases; RET, RET proto‐oncogene; ROS1, ROS proto‐oncogene 1; TKIs, tyrosine kinase inhibitors.

##### Covalent binding site mutation

A less common yet formidable resistance mechanism in targeted therapy is the covalent binding site mutation. As discussed above, the *EGFR*‐T790M mutation can arise in 50−60% of NSCLC patients after receiving treatment with first‐ and second‐generation EGFR‐TKIs.^55^ Osimertinib, a third‐generation EGFR‐TKI with greater potency and selectivity, was designed specifically to overcome the *EGFR*‐T790M resistance mutation through potent covalent‐binding to the ATP‐binding pocket.^93^ In NSCLC patients with T790M acquired resistance mutation treated with first‐generation EGFR‐TKIs, osimertinib showed a superior overall response rate (ORR) (71 vs. 31%) and longer PFS (10.1 vs. 4.4 months) compared with conventional chemotherapy.^94^ Following this, osimertinib was compared with standard first‐ and second‐generation EGFR‐TKIs in patients with advanced‐stage *EGFR*‐positive NSCLC in the pivotal FLAURA trial, revealing a prolonged median PFS (18.9 vs. 10.2 months).^95^

While osimertinib effectively counteracts the *EGFR*‐T790M resistance mutation, its covalent binding to cysteine 797 (C797) residue in the ATP‐binding pocket makes C797 residue vulnerable to mutations.^23^ Unsurprisingly, *EGFR*‐C797S is reported as the most frequent resistance mutation to osimertinib, which has been identified in 10−33% patients after second‐line osimertinib treatment, and 6% patients after first‐line osimertinib treatment.^96^, ^97^, ^98^, ^99^ *EGFR*‐C797S mutation occurs at the *EGFR* C797 codon in exon 20, which is located within the ATP‐binding cleft.^100^, ^101^ The mutation leads to a substitution of cysteine with serine, resulting in the loss of the covalent bond between osimertinib and EGFR.^100^, ^101^, ^102^ Nevertheless, it is noteworthy that the allelic context where C797S arises holds potential implications for treatment. In some circumstances, patients harboring the C797S mutation may remain responsive to quinazoline‐based EGFR‐TKIs.^103^, ^104^, ^105^ When C797S emerges *in trans* with the T790M mutation, patients are responsive to both first‐generation and third‐generation EGFR‐TKIs to address C797S‐ and T790M‐positive alleles, respectively.^103^, ^104^, ^105^ Conversely, when the mutations occur *in cis*, patients demonstrate resistance to all available EGFR‐TKIs, either alone or in combination.^103^, ^104^, ^105^ Similar to *EGFR*‐C797S, *HER2*‐C805S has also been reported to cause resistance to HER2‐TKI therapy in *HER2*‐mutated preclinical cell models.^106^ However, the clinical existence and significance of *HER2*‐C805S covalent mutation is yet to be determined.

##### Compound mutations

Compound mutations refer to the presence of two or more mutations within the same gene or across different genes in the same molecule.^107^, ^108^ Compound mutations have the potential to confer cross‐resistance to multiple TKIs through steric hindrance mechanisms.^107^, ^109^ Compound mutations were first reported in CML patients following sequential therapy with different ABL kinase inhibitors.^108^ In a study, 20 out of 48 CML patients receiving various ABL‐TKI treatments were found to harbor compound mutations.^107^ However, the genuineness of BCR–ABL compound mutations has been long debated due to their rarity of “low‐level mutations,” which can lead to false‐positive results in polymerase chain reaction amplification.^110^, ^111^ By applying next‐generation sequencing (NGS), Deininger et al.^111^ reported that no single or compound mutation was consistently identified to confer primary or acquired resistance to BAR–ABL inhibitor ponatinib in chronic‐phase CML patients. In *EGFR*‐mutant NSCLC, *EGFR* compound mutations are defined as double or multiple mutations occurring at the EGFR tyrosine kinase domain.^112^, ^113^ *EGFR* compound mutations are usually a combination of an *EGFR* typical mutation, such as ex19del, L858R, G719X, and atypical mutations.^113^ It is reported that patients with *EGFR* compound mutations have poorer clinical outcomes than patients with EGFR‐TKI sensitive mutations (ex19del, L858R), but have a more favorable prognosis than patients with resistant mutations (T790M, ex20ins).^112^, ^114^ Similarly, compound *ALK* resistance mutations were identified in *ALK*‐positive patients treated with ALK‐TKIs.^63^ Some studies show that most on‐target *ALK* mutations conferring resistance to third‐generation ALK‐TKI lorlatinib are *ALK* compound mutations.^23^, ^115^, ^116^, ^117^, ^118^, ^119^, ^120^

##### Other site mutations

In addition to the classic secondary mutations discussed above, mutations in other sites of target kinase domains have also been reported to confer TKI resistance. Typically, other site mutations occur in the kinase residues with important functions. They usually are less common. Tyrosine kinase domains exhibit common structural elements, such as the ATP‐binding pocket located between an N‐terminal lobe containing the αC helix, and a C‐terminal lobe containing an activation loop. Mutations in these structural components can cause resistance to inhibitors. For example, *EGFR*‐T854A mutation in the ATP‐binding site, *EGFR*‐D761Y, *EGFR*‐L747S mutations near the αC helix, and *EGFR*‐L792F/H (1.2%) in the hinge region have been found to confer resistance to EGFR‐TKIs.^121^, ^122^, ^123^, ^124^ D276G mutation, located on the β3‐αC loop of *BCR–ABL* kinase domain, was reported to cause resistance to imatinib by inducing destabilization of the inactive conformation of the kinase.^125^ Similarly, ATP‐binding site mutation (*ALK*‐G1269A) and near αC helix site mutations (*ALK*‐C1156Y, *ALK*‐L1152R, *ALK*‐F1174C, and *ALK*‐1151T insertion) were identified in *ALK*‐rearranged cancers, conferring resistance by causing conformational changes.^87^, ^126^, ^127^, ^128^

#### 2.1.2 Amplification of target genes or loss of target mutations (on‐target)

Cancer cells may increase the expression of the target protein, allowing them to maintain signaling despite TKI inhibition. For example, *EGFR* gene amplification has been reported in many studies as a common resistance mechanism in EGFR‐TKI treatment.^129^ *EGFR* amplification was identified in 4.1−10% of patients resistant to osimertinib in second‐line treatment.^129^, ^130^ In addition, wild‐type (WT) *EGFR* amplification, but not the mutant alleles, is proved to be sufficient to confer acquired resistance to third‐generation EGFR‐TKIs in NSCLC.^131^ *MET* gene amplification, overexpression, and constitutive activation are found in cells resistant to MET inhibitors.^132^ *ALK* copy number gain, or amplification is reported in NSCLC patients resistant to crizotinib.^87^, ^133^

Other than *EGFR*‐C797S secondary mutation, *EGFR*‐T790M mutation‐loss represents another major resistance mechanism to osimertinib.^129^ In a study analyzing paired pre‐ and posttreatment samples from 49 NSCLC patients resistant to second‐line osimertinib treatment, 51% (25 out of 49) of these patients exhibited the loss of T790M.^129^ Similarly, half of the patients resistant to second‐line osimertinib had undetectable plasma *EGFR*‐T790M in the AURA3 trial study.^134^ Typically, the loss of T790M is always associated with the co‐occurrence of acquired off‐target resistance mechanisms such as *MET* amplification, *HER2* amplification, *KRAS* mutation, and cell cycle gene alterations.^134^ Approximately one‐third of patients with *EGFR*‐T790M mutation loss are reported to have at least one acquired resistance mechanism.^134^

#### 2.1.3 Activation of alternative signaling pathways (off‐target)

##### Gene amplification

Aside from the alterations of targeted genes themselves, cancer cells can bypass the inhibited signaling pathway by activating other tyrosine kinase receptors that promote cell survival and proliferation (see Table 4). For example, *MET* amplification is the most frequently observed EGFR‐independent mechanism of resistance to EGFR‐TKIs, representing 5−22% of resistant cases after first/second‐generation EGFR‐TKI treatment, 7−15% after osimertinib first‐line treatment, and 5−50% after osimertinib second‐line treatment.^135^, ^136^, ^137^ *MET* encodes the tyrosine kinase receptor for hepatocyte growth factor (HGF). Amplification of *MET* gene or overactivation of MET receptor by HGF leads to activation of downstream signaling cascades, which are shared among tyrosine kinase family receptors (EGFR, HER2, ALK, and RET), such as phosphatidylinositol 3‐kinase (PI3K) /Akt and RAS/RAF/ERK/mitogen‐activated protein kinase (MAPK). By amplifying *MET* gene, cancer cells switch the downstream signaling pathway from the inhibited kinases to the overactivated MET, and then escape from TKI inhibition. *MET* amplification has been proven to mediate gefitinib resistance by activating Erb‐B2 receptor tyrosine kinase 3 (ERBB3)–PI3K/Akt signaling pathway in lung cancer.^136^ MET amplification is also recognized as a resistance driver to RET‐specific inhibitors (selpercatinib), ALK inhibitors (alectinib), and KRAS inhibitors (sotorasib) in NSCLC.^84^, ^138^, ^139^, ^140^ Additionally, the amplification of *HER2* is proposed as a resistance mechanism to EGFR TKIs in NSCLC patients, and anti‐EGFR monoclonal antibody cetuximab in patients with colorectal cancer.^55^, ^137^, ^141^ *EGFR* and *KIT* amplification are reported in patients resistant to ALK inhibitors.^87^ Proto‐oncogene *LYN* overexpression is identified to confer resistance to STI571 in CML.^142^ *FGFR* (*FGFR1*, *FGFR2*, and *FGFR3*) amplification is reported to be a potential resistance mechanism to osimertinib in NSCLC patients.^143^

**TABLE 4:** Parallel bypass activation leading to TKI resistance by activating alternative receptor tyrosine kinases.

Alterations	Cancer types	TKIs	Targeted kinases	Prevalence (%)	References
MET amplification	NSCLC	Gefitinib erlotinib	*EGFR* activated mutations (e.g., ex19del, L858R)	5–22%	^136^, ^137^
		First‐line osimertinib	*EGFR* T790M	16%	^99^
		Second‐line osimertinib	*EGFR* T790M	18%	^134^
		Abivertinib	*EGFR* activated mutations and *EGFR* T790M	3.8%	^144^
		Selpercatinib pralsetinib	*RET* fusions	15%	^84^
		Poziotinib	*EGFR* ex20ins	8.7%	^145^
		Crizotinib	*ALK* fusion	Cases reported *	^146^
		Alectinib	*ALK* fusion	Cases reported *	^147^
		Adagrasib	*KRAS* G12C	Cases reported *	^148^
HER2 amplification	NSCLC	Gefitinib erlotinib	*EGFR* activated mutations (e.g., ex19del, L858R)	13%	^137^
		First‐line osimertinib	*EGFR* T790M	2%	^99^
		Second‐line osimertinib	*EGFR* T790M	5%	^134^
		Abivertinib	*EGFR* activated mutations and *EGFR* T790M	12.5%	^144^
FGFR overactivation/amplification	NSCLC	Gefitinib	*EGFR* activated mutations (e.g., ex19del, L858R)	Preclinical only	^149^
		AZD9291	T790M	Cases reported *	^150^
		Selpercatinib Pralsetinib	*RET* fusions	Cases reported *	^84^
AXL overexpression/amplification	NSCLC	Erlotinib	*EGFR* ex19del	Preclinical only	^151^
		Osimertinib	*EGFR* T790M	Preclinical only	
	BRCA	Lapatinib Erlotinib	*HER2*	Preclinical only	^152^
	HNC	Erlotinib	*EGFR*	Preclinical only	^153^
		Cetuximab	*EGFR*	Preclinical only	^154^
IGF1R activation	NSCLC	EGFR‐TKI WZ4002	*EGFR* T790M	Preclinical only	^155^, ^156^

This table includes selected cancer types and TKIs and does not aim to represent the entirety of all available TKIs in various cancer types.

“Cases reported *” indicates cases with corresponding alterations were reported in case reports, with no available prevalence rate.

Abbreviations: ALK, anaplastic lymphoma receptor tyrosine kinase; AXL, AXL receptor tyrosine kinase; BRCA, breast carcinoma; CRC, colorectal carcinoma; EGFR, epidermal growth factor receptor; ex19del, exon 19 deletion; ex20ins, exon 20 insertion; HER2, human epidermal growth factor receptor 2; HNC, head and neck carcinoma; IGF1R, insulin‐like growth factor 1 receptor; KRAS, kirsten rat sarcoma viral oncogene; NSCLC, non‐small cell lung cancer; PTC, papillary thyroid carcinoma; RET, RET proto‐oncogene; TKIs, tyrosine kinase inhibitors.

##### Aberrant activation of tyrosine kinase downstream signaling pathways

RAS–RAF–MEK–ERK, PI3K/Akt, and JAK/STAT signaling pathways are crucial in receptor tyrosine kinase (RTK) activation.^157^ Cancer cells can acquire resistance to TKIs by developing alterations in downstream components of the inhibited kinases and bypassing the need for upstream stimulation. For example, acquired *KRAS* (G12C) or *BRAF* (G469A, V599E, or V600E) confer resistance to EGFR, MET, or ALK TKIs in NSCLC.^117^, ^143^, ^158^, ^159^, ^160^, ^161^ In these cases, constitutive activation of the RAS–RAF–MEK–MAPK pathways can be directly induced by altered *KRAS* and *BRAF*, without the need for upstream stimulation.^159^, ^162^ Thus, cancer cells escape from the inhibition of TKIs. Similarly, the mutations in encoding *PI3K* can lead to constitutive activation of the *PI3K/Akt* signaling pathway, mediating resistance to MET inhibitors and EGFR‐TKIs.^55^, ^163^ Additionally, *PTEN* loss or mutation also contributes to EGFR‐TKI resistance by activating *Akt*.^98^, ^164^

#### 2.1.4 Histological transformation (off‐target)

Epithelial to mesenchymal transition (EMT), a process by which epithelial cancer cells acquire mesenchymal characteristics (e.g., loss of cell–cell junctions), allows cancer cells to migrate and invade surrounding tissues.^165^ Cancer cells undergoing EMT become less reliant on the targeted pathway and more resistant to apoptosis induced by TKIs.^165^ EMT is proven to be a potential resistance mechanism to erlotinib, osimertinib, and lorlatinib in NSCLC in both clinical and preclinical settings.^55^, ^119^, ^166^, ^167^ The histological transition of EMT typically involves changes in some specific proteins, such as the downregulation of E‐cadherin.^166^ E‐cadherin interacts with EGFR, playing an important role in NSCLC progression.^168^ Reintroduction of E‐cadherin into NSCLC cell lines after undergoing EMT can restore cancer cell sensitivity to gefitinib therapy.^168^ Additionally, activation of Anexelekto (AXL) is shown to play an important role in EMT and TKI resistance. *AXL* activation was found associated with EMT features and conferring acquired resistance to erlotinib in NSCLC cell lines. Genetically or pharmacologically inhibiting *AXL* increased cancer cell sensitivity to erlotinib.^151^ In addition, transforming growth factor‐beta (TGF‐β) secretion triggered by *EGFR* inhibition was also found to promote EMT and confer resistance to EGFR‐TKI treatment by activating the SAMD pathway in NSCLC.^169^

Aside from EMT, NSCLC to small cell lung cancer (SCLC) transformation is another major histological transformation mechanism leading to TKI‐targeted therapy resistance.^170^ Approximately 3−14% of patients with resistance to first‐ or second‐generation EGFR‐TKIs undergo NSCLC to SCLC transformation.^55^, ^137^ When it comes to the third‐generation EGFR‐TKI, this transformation is even more common.^150^ One proposed hypothesis for the mechanism of SCLC transformation is tumor heterogeneity.^170^ It could be possible that patients had tumors with combined histology (both NSCLC and SCLC components) at the time of diagnosis. The SCLC component became dominant after effective TKI treatment. However, gene sequencing of *EGFR* from both pretreatment and posttreatment biopsy samples revealed that transformed SCLC samples retained their mutant EGFR. This suggests posttreatment SCLC samples are most likely transformed from *EGFR*‐mutant NSCLC.

#### 2.1.5 Tumor microenvironment (off‐target)

TKIs have the potential to reshape the tumor microenvironment (TME) by modulating various components, including tumor‐infiltrating immune cells, immunomodulatory stromal cells, cytokines, chemokines, and other factors, such as hypoxia‐inducible factor‐1α (HIF‐1α).^171^, ^172^, ^173^ Increasing evidence suggests that alterations induced by TKI therapy in the TME may contribute to TKI resistance.^174^, ^175^

Tumor‐infiltrating immune cells, including T cells, natural killer (NK) cells, and macrophages, play a dual role in tumor progression and response to TKI therapy. While activated T cells and NK cells can recognize and eliminate tumor cells, the immunosuppressive TME can inhibit their antitumor functions. For example, regulatory T cells and myeloid‐derived suppressor cells secrete immunosuppressive cytokines (TGF‐β and interleukin‐6 [IL‐6]) and inhibit effector T cell function, contributing to TKI resistance.^176^ Additionally, tumor‐associated macrophages (TAMs) can promote angiogenesis, tumor cell invasion, metastasis, and suppression of T immunity in the TME.^177^ Various studies have demonstrated that elevated infiltration of TAMs in solid tumors is often associated with unfavorable clinical outcomes.^178^, ^179^ Within the TME, TAMs can be categorized into two functionally distinct subtypes, namely classically activated macrophages (M1) and alternatively activated macrophages (M2).^180^ M1 macrophages typically display an antitumor phenotype, while M2‐macrophages are commonly considered as pro‐tumor cells.^180^ During TKI treatment, tumor cells induce TAM M2 polarization. M2‐TAMs foster drug resistance through secreting cytokines such as HGF, vascular endothelial growth factor (VEGF), tumor necrosis factor‐alpha, and IL‐6.^177^, ^181^, ^182^, ^183^, ^184^

Stromal cells within the TME, such as cancer‐associated fibroblasts (CAFs) and tumor‐associated endothelial cells, play a critical role in modulating immune responses and promoting tumor survival.^185^ CAFs secrete growth factors, cytokines, and extracellular matrix proteins that support tumor growth and metastasis.^185^, ^186^, ^187^ For instance, stromal cells including fibroblasts could mediate resistance to RAF inhibitors in *BRAF*‐mutant melanoma by secreting HGF and activating MAPK and PI3K signaling pathways in tumor cells.^186^ CAF‐derived SSP1 was found to be a candidate molecule driving resistance to sorafenib/lenvatinib in HCC.^175^ Furthermore, CAFs can induce EMT in tumor cells, leading to increased invasiveness and resistance to TKI therapy.^188^

Cytokines, chemokines, and other factors produced within the TME regulate immune cell recruitment, activation, and function, thereby influencing tumor progression and response to therapy. For example, IL‐6 and TGF‐β promote tumor cell proliferation, survival, and EMT, contributing to TKI resistance.^174^ Additionally, chemokines such as chemokine (C‐C motif) ligand 2 can recruit immunosuppressive myeloid cells to the TME, further exacerbating immune evasion and TKI resistance.^176^, ^189^, ^190^ In solid tumors, the TME is always characterized by hypoxia, leading to the upregulation of HIF‐1α in tumor cells.^191^ HIF‐1α is a pivotal regulator that can activate the transcription of genes associated with tumor angiogenesis and cell survival, such as VEGF and its receptor VEGFR.^191^ As VEGFR and EGFR share common downstream signaling pathways, elevated VEGF and VEGFR expression contribute to the emergence of acquired resistance to EGFR‐TKIs in NSCLC.^192^ Hypoxia was found to induce resistance to gefitinib in *EGFR*‐mutant NSCLC by activating WT EGFR through the upregulation of TGFα.^193^

### 2.2 Primary or intrinsic resistance

Primary resistance, defined as the lack of initial responses to TKIs, has been observed in 20−30% of advanced *EGFR*‐mutant NSCLC patients,^95^ 20−40% advanced *ALK*‐positive NSCLC patients,^194^ 10% GIST patients,^195^ and other cancer patients.^196^ Primary resistance to TKIs is often associated with coexistence of additional genetic alterations together with TKI‐sensitive mutations within the context of tumor heterogeneity.^197^ Specifically, molecular mechanisms contributing to primary or intrinsic resistance include the presence of TKI‐resistant mutations (e.g., existence of de novo *EGFR*‐T790M and *EGFR* ex20ins are associated with primary resistance to early‐generation EGFR‐TKIs^29^, ^198^), concurrent alterations in other oncogenes (e.g., de novo *MET* amplifications or *KRAS* mutation in EGFR‐TKI resistant patients and *KRAS* mutation in imatinib resistance GIST patients^195^, ^199^, ^200^, ^201^), mutations in downstream signaling pathways (e.g., *PTEN*‐loss confers resistance to EGFR‐TKIs^202^), and germline deletions of gene encoding BCL‐2‐like protein 11 (BIM).^203^

## 3 OVERCOMING STRATEGIES FOR RESISTANCE TO TKI‐TARGETED THERAPY

**FIGURE 3:**
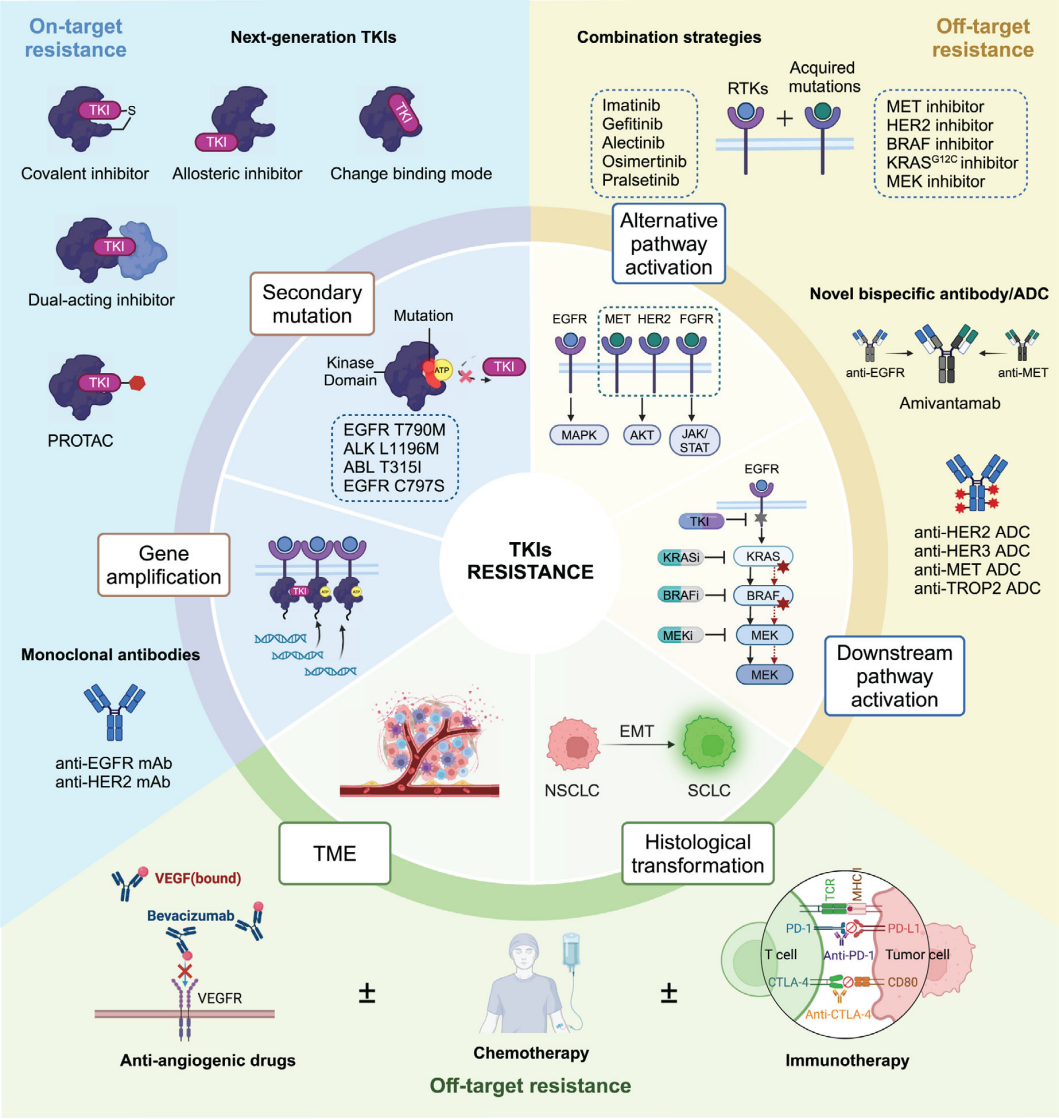
Mechanisms of resistance to TKI and potential strategies of treatments to overcome resistance. ADC, antibody–drug conjugate; mAb, monoclonal antibody; PROTAC, proteolysis targeting chimera; RTKs, receptor tyrosine kinases; SCLC, small cell lung cancer; TME, tumor microenvironment; TKI, tyrosine kinase inhibitor. This figure is created with BioRender.com.

“One‐drug, one‐target, one‐disease” has achieved tremendous success in the past 20 years and forms the basis of tumor precision therapy. However, as our research into tumor resistance mechanisms deepens, we have found that aside from tumors developing acquired resistance due to the target itself, most mechanisms are unrelated to the original target of the drug. So far, considerable progress has been made in developing strategies to prolong the duration of treatment benefits to patients. Examples include develop novel TKIs with broader specificity or improved binding affinity. Target‐specific mutations that confer resistance to first‐line therapies or inhibit multiple signaling pathways simultaneously to prevent bypass mechanisms. Other tumor resistance mechanisms unrelated to the drug target often require combination therapy strategies to overcome. In the following section, new chemical strategies that are based on a different principle to address the emergence of drug resistance are discussed (Figure 3).

### 3.1 Development of next‐generation TKIs

Acquired drug resistance mutations, including gatekeeper, solvent‐front mutations, and aspartic–phenylalanine–glycine (DFG)‐loop mutations, most commonly affect the binding of the drug to its target. Developing new TKIs or finding new sites of action is a promising solution for “on‐target” resistance. For instance, resistance to first‐generation EGFR‐TKIs is commonly associated with the *EGFR* T790M secondary mutation.^55^, ^204^ Osimertinib (Tagrisso) is a third‐generation TKI designed to target the *EGFR* T790M to overcome the resistance of first‐generation EGFR‐TKIs.^205^ The common strategies to overcome resistance mainly include drug structure optimization, designing covalent inhibitors, and designing allosteric inhibitors.

#### 3.1.1 Drug structure optimization

Drug structure optimization is one of the commonly used strategies to overcome tumor resistance and is also an important means for discovering many best‐in‐class drugs. By analyzing the cocrystal structure of mutant proteins and small molecules, compound structures can be continuously optimized based on the original small molecule to obtain completely new small molecule compounds with distinct binding modes.^206^ An important example that demonstrates the clinical impact of this strategy is the ALK inhibitors developed to tackle gatekeeper mutation to crizotinib. *ALK* fusion‐positive NSCLC patients treated with the first‐generation ALK inhibitor crizotinib achieve an objective response rate of 60%, with a PFS of 8–10 months, and significantly prolonged OS.^207^, ^208^ However, many patients develop resistance to crizotinib approximately 1 year after treatment due to the *ALK* L1196M “gatekeeper” mutation.^127^, ^133^, ^209^ Cocrystal structures indicate that introducing methionine at the gatekeeper position 1196 likely adversely impacts the conformational changes in the ATP‐binding entrance and hinge region, affecting crizotinib binding through steric interference.^126^ To overcome L1196 gatekeeper mutation, second‐generation ALK inhibitors ceritinib and brigatinib with a different binding mode compared with that of crizotinib were designed.^126^, ^210^, ^211^ However, although these agents had improved potency and targeted key resistance mutations, they can only block a subset of resistance‐conferring mutant alleles in *ALK* kinase due to partially overlapped binding modes. G1202R was the major resistance mutation site of the second‐generation ALK‐TKIs.^63^, ^212^, ^213^ Therefore, the third‐generation ALK inhibitor lorlatinib has emerged. Compared with the previous two generations of ALK inhibitors, lorlatinib belongs to the macrocyclic drug class with a certain rigid structure within the molecule.^214^ It binds more tightly to the active pocket of the *ALK* protein, thus overcoming resistance caused by mutations such as *ALK* L1196M and G1202R.^63^, ^215^

Therefore, although the above three generations of ALK inhibitors all bind to the active site of ALK protein, they can overcome resistance by utilizing different inhibitor‐amino acid residue contact modes. However, not all types of resistance targets are worthy of being developed as new drug targets. Some resistant mutations may become sensitive again to previous generations of drugs targeting the same target. *ALK* L1198F substitution confers resistance to lorlatinib through steric interference with drug binding. However, L1198F paradoxically enhances binding to first‐generation ALK‐TKI crizotinib, resensitizing resistant cancers to crizotinib.^115^, ^120^, ^209^ In conclusion, the experience with the development of different ALK inhibitors suggests that designing drugs with different binding modes can help overcome resistance to targeted therapy.

#### 3.1.2 Covalent inhibitors

Another chemical strategy to overcome resistance is to design covalent inhibitors. Covalent bonds are stronger than hydrogen bonds and hydrophobic interactions, and covalent small molecules have a higher affinity with the target protein. This approach depends on the nucleophilic amino acid residues (such as cysteine and lysine) in the active pocket of the target protein to form covalent bonds with electrophilic moiety in inhibitors.^216^, ^217^

The most typical example of covalent inhibitors is the design of EGFR inhibitors. Represented by gefitinib and erlotinib, the first‐generation EGFR inhibitors are noncovalent ATP‐competitive inhibitors of WT EGFR and have shown good efficacy in NSCLC patients with the *EGFR* L858R/19del mutation.^218^, ^219^, ^220^ However, after 8–14 months of treatment with the first‐generation EGFR inhibitors, about 50% of patients develop the T790M resistance mutation at the gatekeeper site of the *EGFR* kinase domain.^55^, ^57^, ^204^, ^221^, ^222^ Unlike the analogous L1196M gatekeeper mutation in the *ALK* kinase domain mentioned above, which introduces a steric impediment for crizotinib binding,^126^ *EGFR* T790M only modestly affects gefitinib or erlotinib binding. However, more importantly, it significantly increases its affinity for ATP, similar to that of WT *EGFR*.^58^ Competitive noncovalent inhibitors like gefitinib lack sufficient affinity for *EGFR* T790M to effectively compete with ATP, leading to treatment failure. Cocrystal structure studies have revealed a cysteine residue at position C797 in the ATP‐binding site of EGFR. Small molecules that covalently bind to C797 can effectively inhibit the kinase activity of *EGFR* T790M.^205^, ^206^, ^223^, ^224^, ^225^ Based on this strategy, the second‐generation irreversible covalent inhibitors afatinib and dacomitinib were designed to enhance potency and try to overcome the resistance limitations of *EGFR* T790M. However, due to the ATP affinity of *EGFR* T790M being similar to WT *EGFR*, second‐generation covalent inhibitors can cause strong adverse reactions, especially skin rash and diarrhea, and limit the ability to achieve plasma concentrations sufficient to inhibit *EGFR* T790M.^224^, ^226^, ^227^ This challenge led to embark on innovative strategies to develop third‐generation reversible inhibitors osimertinib that were specifically designed to target the *EGFR* T790M mutant, while maintaining activity against the initial activating mutations and retaining sufficient selectivity over WT *EGFR* to address the limitations of afatinib and dacomitinib.^205^ These studies indicate that designing covalent compounds targeting cysteine residues in the active site is feasible and can overcome resistance to prior‐generation TKIs.

#### 3.1.3 Allosteric inhibitors

Most approved TKIs, whether in clinical use or in preclinical development, are ATP‐competitive inhibitors targeting the ATP‐binding pocket of kinases.^228^ Based on the highly conserved conformation of kinase activation loop starting with DFG, ATP‐competitive inhibitors are classified into type I and type II inhibitors. Type I inhibitors (such as gefitinib) bind in the “DFG‐in” conformation within the ATP‐binding pocket in its active state, while type II inhibitors (such as imatinib) bind to the hinge region and allosteric site of ATP pocket in the inactive state.^229^, ^230^, ^231^ However, the conservation of the ATP‐binding pocket among different kinases poses a challenge for the development of highly selective kinase inhibitors. Additionally, the efficacy of ATP‐competitive or covalent inhibitors can be compromised by active site mutations that can arise upon prolonged drug treatment.^96^, ^232^, ^233^ One strategy is to target kinase allosteric pockets outside the ATP‐binding site. Such allosteric modulators bind to sites that are less conserved than ATP‐binding sites and only accessible upon conformational changes, providing various advantages such as higher selectivity and extended drug target residence times.^228^, ^234^

A good example of the enhanced clinical impact of allosteric inhibitors is provided by ABL1 kinase. The first‐in‐class BCR–ABL inhibitor imatinib induced resistance in CML through the gatekeeper T315I mutation.^235^, ^236^ Asciminib, targeting the allosteric pocket of ABL1 kinase, can effectively block the kinase activity of ABL1 T315I mutation, overcoming resistance to ATP‐competitive inhibitors like imatinib.^237^, ^238^ Allosteric inhibitors mimic the natural regulatory mechanisms of protein kinases, providing very high specificity. Therefore, targeting the “allosteric pocket” on the target protein instead of the “orthosteric pocket” is one effective strategy to overcome tumor resistance.

### 3.2 Combination therapies

The development of next‐generation inhibitors to overcome drug resistance has become entrenched in current kinase drug discovery. However, as exemplified by osimertinib and lorlatinib, continual development of further next‐generation agents that directly target the drug‐resistant kinase mutant still acquired resistance.^96^, ^213^, ^215^, ^221^ Besides, resistance caused by “on‐target” mutations only accounts for a minority of cases. The majority of patients develop resistance due to “off‐target” pathway activation, while some resistance mechanisms remain unknown. Only a small proportion of patients can continue with monotherapy, while more patients require combination therapy to simultaneously target kinase protein and off‐target mutations. Combination therapies have the potential to suppress the emergence of drug resistance by completely and simultaneously suppressing cooperating oncogenic signals or concurrently blocking bypass signaling pathways that may mediate drug resistance. In the following section, we highlight recent advances in rational combination strategies that are based on different principles. Some of these drug combinations are already in clinical practice, whereas others are in earlier stages of clinical development.

#### 3.2.1 Combination therapies with targeted agents

As mentioned above, parallel or downstream activation of oncogenic signaling can reduce the efficacy of primary target inhibition by reactivating the signaling pathway. Such as BRAF or *KRAS* mutations in EGFR‐TKI‐treated lung cancer, or *MEK* mutations in BRAF inhibitor resistant malignant melanoma.^143^, ^160^, ^239^, ^240^ One approach involves dual inhibition of oncogenic signaling, either upstream or downstream of the driver gene, by inhibiting multiple nodes of a signaling pathway, is a rational and effective strategy to overcome drug resistance. Examples of this strategy are the combination of BRAF and MEK inhibitors to delay resistance in malignant melanoma. Reactivation of the MAPK pathway is the dominant acquired resistance mechanism of BRAF inhibitors.^241^ The combination of BRAF and MEK inhibitors (e.g., dabrafenib and trametinib) is now a standard regimen for *BRAF*‐mutant melanoma.^242^, ^243^ Src homology 2 domain‐containing protein tyrosine phosphatase 2 (SHP2) is a central downstream effector of many RTKs activation signal cascades.^244^ Preclinical studies have shown that inhibiting SHP2 can suppress MAPK pathway activation and inhibit tumor growth dependent on RTK pathway activation and/or carrying oncogenic *KRAS* mutations.^245^, ^246^, ^247^, ^248^ Therefore, SHP2 inhibitors as part of combination therapy may significantly enhance the therapeutic effects of RTK, RAS, and MAPK pathway inhibitors (e.g., EGFR‐TKIs, KRAS^G12C^‐TKIs).

Another promising approach is the use of combination therapy that combines inhibitors of the original kinase mutation with inhibitors of a parallel kinase to block the predominant cause of bypass resistance. For example, *MET* amplification is one of the most frequent mechanisms of acquired resistance to EGFR‐TKIs. *MET* amplification‐dependent resistance is caused by persistent activation of signaling pathways downstream of *EGFR*, such as those mediated by MAPK, signal transduction and activator of transcription, and PI3K–Akt, which bypass EGFR signaling. Several clinical evidence demonstrated that combining an EGFR‐TKI with a MET inhibitor may overcome *MET*‐mediated resistance.^249^, ^250^ Savolitinib is an oral, potent, and highly selective MET‐TKI. In the phase Ib TATTON trial, MET‐positive patients with disease progression following osimertinib, treatment with osimertinib plus savolitinib yielded an ORR of 67%, with a median duration of response of 12.4 months.^249^ Tepotinib, another oral selective MET‐TKI, in combination with osimertinib is under investigation in the INSIGH2 trial (NCT03940703). PFS and OS were longer with tepotinib plus gefitinib than with chemotherapy in patients with high MET overexpression or *MET* amplification.^250^ Similarly, dual ALK‐MET inhibition may also overcome *ALK*‐positive lung cancer with *MET*‐driven resistance.^251^, ^252^ Clinical trials of TKI combination therapy based on off‐target resistance mechanisms are summarized in Table 5.

**TABLE 5:** Clinical trials of TKIs combination therapy based on off‐target resistance mechanisms.

Target	Phase	Clinical trial number	TKIs	Combination	Treatment arms	Trial population	Results	References
EGFR	II	NCT03778229 (SAVANNAH)	Osimertinib	MET inhibitor	Osimertinib+savolitinib	*EGFR*‐mutant, *MET*‐driven NSCLC progressed on osimertinib	IHC90+ and/or FISH10+: ORR: 49% mDOR: 9.3 m mPFS: 7.1 m	^256^
	Ib	NCT02143466 (TATTON)	Osimertinib	MET inhibitor	Part B: osimertinib+savolitinib	*MET*amp, *EGFR*‐mutated NSCLC, and progression on prior EGFR‐TKI	ORR: 30% (21/69, all PR) DCR: 75% (52/69) mDOR: 7.9months mPFS: 5.4 months	^257^
	II	NCT03944772 (ORCHARD)	Osimertinib	*MET* inhibitor, First‐generation EGFR‐TKI, anti‐EGFR mAbs, CT plus ICIs	*MET* alterations: osimertinib+savolitinib *EGFR* C797X:Osimertinib+gefitinib *EGFR* alterations:osimertinib+necitumumab *ALK* rearrangement: osimertinib+alectinib *RET* rearrangement: osimertinib+selpercatinib Biomarker nonmatched: durvalumab+pemetrexed +carboplatin	*EGFR*‐mutant NSCLC progressed on osimertinib	MET alterations: ORR: 41% (7/17, all PR) DCR: 82% (14/17)	^258^
	Ib	NCT02374645	Gefitinib	MET inhibitor	Gefitinib+savolitinib	*EGFR*‐mutant Chinese NSCLC progressed on prior EGFR‐TKI	In *EGFR* T790M‐negative: ORR: 52% (12/23) mDOR: 7.2 m mPFS: 4.2 m	^259^
	II	NCT03940703 (INSIGHT‐2)	Osimertinib	MET inhibitor	Osimertinib+tepotinib	*MET*amp NSCLC and first‐line osimertinib resistance	Interim data: 98 pts with FISH *MET*amp: ORR: 43.9% mDOR: 9.7 m mPFS: 5.4 m 31 pts with NGS *MET*amp: ORR: 51.6% mDOR: 5.6 m mPFS: 4.6 m	
	Ib/II	NCT01982955 (INSIGHT)	Gefitinib	MET inhibitor	Gefitinib+tepotinib vs. CT	*MET* overexpression (IHC2+ or 3+) or *MET* NSCLC progressed on EGFR‐TKI	In *EGFR* T790M−: ORR: 67 vs. 43% mDOR: 19.9 vs. 2.8 m mPFS: 16.6 vs. 4.2 m mOS: 37.3 vs. 13.1 m	^250^
	Ib/II	NCT01610336	Gefitinib	MET inhibitor	Gefitinib+capmatinib	*EGFR*‐mutated, *MET*‐amplified/overexpressing NSCLC progressed on EGFR‐TKI	ORR: 47% (17/36, all PR) DCR: 75% (27/36) mPFS: 5.5 m	^260^
	Ib	NCT05430386	Almonertinib	MET inhibitor	Almonertinib+HS‐10241	*MET*‐amplified NSCLC progressed after EGFR‐TKI	8 PR and 4 SD among 13 pts	^261^
	Ib/II	NCT02335944	Nazartinib	MET inhibitor	Nazartinib+capmatinib	*EGFR*‐mutant NSCLC (post‐EGFR‐TKI and treatment naïve)	*MET*+: ORR 43.5% *MET*−: ORR 27.9% TN: ORR 61.7%	^262^
	III	NCT04988295 (MARIPOSA‐2)	Lazertinib	EGFR/MET bispecific antibody	Lazertinib+amivantamab+CT amivantamab+CT CT	Osimertinib‐relapsed *EGFR*‐mutant NSCLC	Amivantamab+CT Vs. lazertinib+amivantamab +CT vs. CT: ORR: 64 vs. 63 vs. 36% mPFS: 8.2 vs. 8.3 vs. 4.2 m	^263^
	I	NCT02609776 (CHRYSALIS)	Lazertinib	EGFR/MET bispecific antibody	Lazertinib+amivantamab	Osimertinib‐relapsed *EGFR*‐mutant NSCLC	ORR: 36% mDOR: 9.6 m CBR ≥ 11 weeks: 64% mPFS: 4.9 m	^264^
	I/II	NCT03784599 (TRAEMOS)	Osimertinib	Anti‐HER2‐ADC	Trastuzumab‐emtansine+osimertinib	*EGFR*‐mutated NSCLC, progressing on osimertinib and *HER2* overexpression	ORR: 4% (1 of 27) mPFS: 2.8 m	^265^
	Ib	NCT04001777	Osimertinib	Bcl‐2 family protein inhibitor	Pelcitoclax(APG1252) +osimertinib	Patients with third‐generation EGFR TKI‐resistant NSCLC	3 PR of 20 pts, including 2 pts with osimertinib‐resistant NSCLC	^266^
	I	NCT03891615	Osimertinib	PARP inhibitor	Osimertinib+niraparib	NSCLC with *EGFR* mutation progression on osimertinib	Ongoing	–
	I	NCT03516214 (EATON)	Nazartinib	MEK inhibitor	Nazartinib+trametinib	Advanced NSCLC harboring *EGFR* del 19 or p.L858R, first‐line or after failure of any EGFR TKI	Ongoing	–
	I	NCT01570296	Gefitinib	PI3K inhibitor	Gefitinib+BKM120	Patient Group 1: EGFR TKI Resistant; Patient Group 2: activated *PI3K* status and overexpress *EGFR*	Ongoing	–
	Ia	NCT06032936	Osimertinib	SHP2 inhibitor	Osimertinib+BBP‐398	NSCLC pts with *EGFR* mutations and with previously third‐generation EGFR‐TKIs treated or EGFR‐TKI naïve	Ongoing	–
ALK	Ib/II	NCT02292550	Ceritinib	CDK4/6 inhibitor	Ceritinib+ribociclib	Stage IIIB/IV *ALK*+ NSCLC	ORR was 50% (4/8)in ALKi‐naïve pts; 64% (9/14) in pts with prior crizotinib; 0% (0/5;) in pts with prior third‐gen ALKi.	^267^
	I	NCT03087448	Ceritinib	MEK inhibitor	Ceritinib+trametinib	Patients with refractory NSCLC harboring *ALK* or *ROS1* fusions	ORR: 22% DCR: 56% mDOR: 7.85 m mPFS: 3 m	^268^
	Ib/II	NCT03202940	Alectinib	MEK inhibitor	Alectinib+cobimetinib	*ALK*+ NSCLC pts progressed on alectinib	Ongoing	–
	I/II	NCT05845671	–	EGFR/MET bispecific antibody	ALK/ROS1/RET‐TKI+amivantamab	NSCLC harboring *ALK*, *ROS1*, and *RET* Gene Fusions progression on at least one prior TKI	Ongoing	–
	Ib	NCT04005144	Ceritinib	Anti‐PD‐1 mAb	Ceritinib+nivolumab	ALK‐TKI naïve and pretreated NSCLC	ALK‐TKI pretreated: ORR: 50% mPFS: 6.4 m	^269^
	I	NCT04777084	Lenvatinib	Bispecific anti‐PD‐1/​PD‐L1 antibody	Lenvatinib+IBI318	Cohort B: advanced NSCLC with *EGFR*‐sensitive mutation/*ALK* fusion after EGFR‐TKI/ALK‐TKI resistance.	Ongoing	–
	II	NCT05456256 (HARMONIC)	–	*EGFR*, *ALK*, *MET*, and *ROS1*	LP‐300+carboplatin+ pemetrexed	Patients who are never smokers with lung adenocarcinoma and have relapsed after treatment with tyrosine kinase inhibitors	Ongoing	–
KRAS	I/II	NCT03785249	Adagrasib	SHP2 inhibitor	Adagrasib+TNO155	Pts with advanced solid tumors harboring a *KRAS* G12C mutation (excluding NSCLC and CRC)	ORR: 35.1% mDOR: 5.3 m mPFS: 7.4 m	^270^
	Ib	NCT04185883	Sotorasib	SHP2 inhibitor	Sotorasib+RMC4630	Pts with *KRAS* G12C‐mutated NSCLC, CRC, or other solid tumors	Pretreated: ORR 27% KRAS G12C naïve: ORR 50%	^271^
	I/II	NCT05288205	JAB21822	SHP2 inhibitor	JAB21822+JAB3312	Solid tumors harboring *KRAS* G12C mutation	Ongoing	–
	I/II	NCT05375994 (RAMP 204)	Adagrasib	MEK/RAF inhibitor	Adagrasib+VS6766	Pts with *KRAS* G12C‐mutant NSCLC	Ongoing	–
	I/II	NCT04793958	Adagrasib	Anti‐EGFR mAb	Adagrasib monotherapy Adagrasib+cetuximab	Pts with metastatic colorectal cancer with mutant *KRAS* G12C	Adagrasib monotherapy ORR: 23% mDOR: 4.3 m mPFS: 5.6 m Adagrasib+cetuximab: ORR: 46% mDOR: 7.6 m mPFS: 6.9 m	^272^
	I/II	NCT05002270 NCT05194995	JAB21822	Anti‐EGFR mAb	JAB21822+Cetuximab	Solid tumors CRC	Ongoing	–
	I/Ib	NCT04975256	Adagrasib	Pan KRAS/SOS1 inhibitor	Adagrasib+BI 1701963	Pts with advanced solid tumors haboring *KRAS* G12C mutation	Ongoing	–
	I/Ib	NCT05178888 (KRYSTAL‐16)	Adagrasib	CDK4/6 inhibitor	Adagrasib+pabociclib	Pts with solid tumors harboring a *KRAS* G12C mutation previously treated with at least 1 standard therapy		
BCR–ABL	II	NCT03610971	BCR‐ABL TKI	JAK1/2 inhibitor	*BCR–ABL* TKI+ruxolitinib	CML pts relapsed after a prior attempt at TKI discontinuation	Ongoing	–
	I/II	NCT01914484	Nilotinib	JAK1/2 inhibitor	Nilotinib+ruxolitinib	Pts with Philadelphia‐positive CML or ALL who is resistant to *BCR–ABL* inhibitor	Ongoing	–

Abbreviations: ALK, anaplastic lymphoma receptor tyrosine kinase; ALL, acute lymphoblastic leukemia; Bcl‐2, B‐cell lymphoma‐2; CBR, clinical benefit rate; CDK, cyclin‐dependent kinase; CML, chronic myeloid leukemia; CRC, colonrectal cancer; CT, chemotherapy; DCR, disease control rate; EGFR, epidermal growth factor receptor; FISH, fluorescence in situ hybridization; HER2, human epidermal growth factor receptor 2; ICIs, immune checkpoint inhibitors; IHC, immunohistochemical; JAK, Janus kinase; KRAS, kirsten rat sarcoma viral oncogene; mAb, monoclonal antibody; mDoR, median duration of response; MEK, mitogen‐activated protein kinase kinase; MET, mesenchymal–epithelial transition factor; METamp, MET gene amplification; mOS, median overall survival; mPFS, median progression‐free survival; NSCLC, non‐small cell lung cancer; ORR, overall response rate; PARP, poly‐ADP‐ribosepolymerase; PD‐1, programmed death‐1; PD‐L1, programmed death‐ligand 1; pts, patients; ROS1, c‐ros oncogene 1 receptor tyrosine kinase; SHP2, Src homology phosphotyrosyl phosphatase 2; SOS, son of sevenless; TKI, tyrosine kinase inhibitor; TN, treatment naïve.

In addition, combining drugs targeting the same protein kinase but with different binding modes can effectively address drug resistance issues. For example, the combination of the BCR–ABL1 kinase inhibitor dasatinib, which targets the ATP‐binding active site, with the allosteric inhibitor asciminib, can significantly overcome resistance to dasatinib and even achieve tumor regression.^253^ Designing multitarget inhibitors against newly emerging resistant targets also belongs to combination therapy in broad terms. In recent years, cyclin‐dependent kinase 4/6 (CDK4/6) inhibitors have made significant breakthroughs in the treatment of advanced or metastatic breast cancer. However, with the widespread clinical use of these drugs, tumor resistance has emerged as a challenge. Resistance to CDK4/6 inhibitors is mainly triggered by the activation of MYC proto‐oncogene protein (MYC), G1/S‐specific cyclin‐E1 (CCNE1), and CDK2.^254^ Therefore, the combination of CDK4/6 inhibitors with CDK2 inhibitors may overcome resistance to CDK4/6 inhibitors in patients. PF3600 is the first known CDK2/4/6 small molecule inhibitor in clinical development and offers a new approach to overcoming resistance to CDK4/6 inhibitors.^255^

#### 3.2.2 Antiangiogenic drugs combinations

Combining targeted therapies with antiangiogenic drugs is a new therapeutic approach currently being explored, most notably in the first‐line setting, with mixed results to date (Table 6). Antiangiogenic‐targeted drugs, represented by bevacizumab and anlotinib, have become indispensable treatment options for a variety of cancers as they can inhibit tumor angiogenesis, promote normalization of tumor blood vessels, and enhance drug delivery.^273^ Combination therapy with antiangiogenic drugs and immune checkpoint inhibitors (ICIs) or chemotherapy has emerged as a potential strategy to enhance the therapeutic efficacy of cancer.^274^, ^275^ In recent years, one of the prevalent research directions is the combination of angiogenic inhibitors with targeted therapies to overcome resistance to TKIs. Preclinical studies have indicated that simultaneous blockade of the VEGF pathway has synergistic antitumor activity and may delay the development of resistance to EGFR‐TKIs or ALK‐TKIs.^276^, ^277^, ^278^ JO25567, NEJ026, CTONG1509, and RELAY studies have shown that the combination of EGFR‐TKIs with antiangiogenic inhibitors (e.g., bevacizumab, anlotinib, ramucirumab) may have prolonged PFS in advanced EGFR‐mutant NSCLC compared with EGFR‐TKIs monotherapy, although these studies have not yielded OS benefit.^279^, ^280^, ^281^, ^282^ The effectiveness of combining antiangiogenic therapy with EGFR‐TKIs to enhance efficacy is still controversial. The relevant mechanisms of action are not yet fully understood. Additionally, there is no evidence to suggest that antiangiogenic agents may help EGFR‐TKI overcome known resistance mechanisms. In phase I studies of bevacizumab plus osimertinib and ramucirumab plus osimertinib, the summarized resistance mechanisms were not significantly different from those of monotherapy.^283^, ^284^ This comparison indirectly suggests that antiangiogenic agents do not help overcome osimertinib monotherapy resistance mechanisms, although we cannot completely rule out the possibility of delaying or reducing the occurrence of osimertinib resistance. Furthermore, the risk of adverse events is higher with combination therapy, with hypertension and proteinuria being particularly noticeable.^285^ Similarly, the potential benefit from an antiangiogenic agent combined with ALK‐TKI in NSCLC patients remains to be determined. In phase I/II small cohort trials, the combination of alectinib and bevacizumab has been demonstrated to be tolerable. However, there was no significant improvement in efficacy.^286^, ^287^ To gain further insight and guide future studies, additional translational research and deeper mining of existing studies are necessary.

**TABLE 6:** Clinical trials of combination therapy of TKIs and antiangiogenic drugs.

Target	Phase	Clinical trial number	TKIs	Combination	Treatment arms	Trail population	Results	References
EGFR	III	NCT04028778 (FL‐ALTER)	Gefitinib	MKIs targeting VEGFRs, FGFRs, PDGFRs, c‐kit and MET	Getinib+anlotinib Gefitinib+placebo	First‐line treatment of NSCLC with an EGFR 19del or 21 L858R mutation	mPFS: 14.75 m vs. 11.20 m ORR: 76.13 vs. 64.52% mDOR: 12.48 vs. 9.46 m	^288^
	II	ALTER‐L004	Icotinib	MKIs targeting VEGFRs, FGFRs, PDGFRs, c‐kit and MET	Icotinib+anlotinib	First‐line treatment option for advanced NSCLC carrying EGFR mutation with or without concurrent mutations	ORR: 68.5% DCR: 98.2% mPFS: 15.1 m mDOR: 13.5	^289^
	Ib/IIa	NCT04770688 (AUTOMAN)	Osimertinib	MKIs targeting VEGFRs, FGFRs, PDGFRs, c‐kit and MET	Osimertinib+anlotinib	Treatment‐naïve patients of locally advanced or metastatic nonsquamous EGFR‐mutated NSCLC	ORR: 65.2% DCR: 95.7%	^290^
	II	ALWAYS	Aumolertinib	MKIs targeting VEGFRs, FGFRs, PDGFRs, c‐kit and MET	Aumolertinib+anlotinib	First‐line treatment of advanced NSCLC patients with EGFR mutations.	ORR: 96.15% DCR: 100% CNS ORR: 76.47% CNS DCR: 100%	^291^
	II	NCT02803203	Osimertinib	Anti‐VEGF mAb	Osimertinib+bevacizumab	First‐line treatment of advanced NSCLC patients with EGFR mutations.	ORR: 69% PFS‐12 months: 0.70	^292^
	II	JO25567	Erlotinib	Anti‐VEGF mAb	Erlotinib+bevacizumab Erlotinib	Stage IIIB/IV or recurrent nonsquamous NSCLC with EGFR mutations	mPFS: 16.0 vs. 9.7 m	^282^
	III	NEJ026	Erlotinib	Anti‐VEGF mAb	Erlotinib+bevacizumab Erlotinib	Stage IIIB/IV or recurrent nonsquamous NSCLC with EGFR mutations	mPFS: 16.9 vs. 13.3 m	^281^
	III	RELAY	Erlotinib	Anti‐VEGFR2 mAb	Erlotinib+ramucirumab Erlotinib+placebo	Pts with untreated EGFR‐mutated metastatic NSCLC	PFS: 19.4 vs. 12.4 m	^279^
	II	NCT03909334 (RAMOSE)	Osimertinib	Anti‐VEGFR2 mAb	Osimertinib+ramucirumab Osimertinib	Treatment‐naïve EGFR‐mutant NSCLC	PFS: 24.8 vs. 15.6 m ORR: 76.3 vs. 80.4% DCR: 96.8 vs. 95.7%	^293^
ALK	I/II	NCT02521051	Ceritinib	Anti‐VEGF mAb	Alectinib+bevacizumab	Pts with advanced ALK‐rearranged NSCLC	3(60%) of 5 ALK TKI‐pretreated patients had objective responses; mPFS: 9.5 m	^286^
	I/II	NCT06007937	Loratinib	Anti‐VEGFR2 mAb	Lorlatinib+ramucirumab	Treatment‐naïve or progressed of at least one second‐generation ALK TKI	Ongoing	–
	Ib	NCT04227028	Brigatinib	Anti‐VEGF mAb	Brigatinib+bevacizumab	ALK+ NSCLC pts previously progressed on prior ALK‐directed therapy	Ongoing	–
	II	ACTRN12622000973718(SHERLOCK)	Sotorasib	Anti‐VEGF mAb	Sotorasib+ bevacizumab+carboplatin‐pemetrexed	First line treatment of nonsquamous advanced NSCLC with KRAS G12C mutation	Ongoing	–

Abbreviations: ALK, anaplastic lymphoma receptor tyrosine kinase; ALL, acute lymphoblastic leukemia; CBR, clinical benefit rate; CML, chronic myeloid leukemia; CRC, colorectal cancer; DCR, disease control rate; EGFR, epidermal growth factor receptor; FGFR, fibroblast growth factor receptor; mAb, monoclonal antibody; mDoR, median duration of response; MET, mesenchymal–epithelial transition factor; METamp, MET gene amplification; MKIs, multiple kinase inhibitors; mOS, median overall survival; mPFS, median progression‐free survival; NSCLC, non‐small cell lung cancer; ORR, overall response rate; PDGFR, platelet‐derived growth factor receptor; pts, patients; TKI, tyrosine kinase inhibitor; TN, treatment naïve; VEGF, vascular endothelial growth factor; VEGFR, vascular endothelial growth factor receptor.

#### 3.2.3 Chemotherapy‐based combinations

Chemotherapy remains the cornerstone of treatment for advanced‐stage cancer. Particularly for patients with resistance to targeted therapy, especially when the resistance mechanism is unclear and targeted therapy is no longer applicable, chemotherapy remains the standard treatment recommended by guidelines. The results of the FLAURA2 study revealed that the median PFS in the osimertinib plus chemotherapy group was significantly longer than that in the osimertinib monotherapy group in first‐line treatment (25.5 vs. 16.7 months, hazard ratio 0.62). OS data are currently immature, but they indicate a trend toward benefit.^294^ The combination of the third‐generation BCR–ABL1 inhibitor olverembatinib with the Bcl‐2 inhibitor venetoclax has shown high efficacy in relapsed Ph+ acute lymphoblastic leukemia (ALL) patients. A phase II clinical study combining these two drugs with low‐dose chemotherapy demonstrated remarkable efficacy and safety.^295^ The combination of platinum‐based chemotherapy with PARP inhibitors has shown promising efficacy in the treatment of ovarian cancer or breast cancer with BRCA1/2 mutations.^296^, ^297^ At present, most clinical studies of targeted therapy combined with chemotherapy focus on first‐line treatment, some combination regimens have shown certain advantages in certain cancer types. Tumor driver genes typically exhibit strong oncogenicity, leading to a high dependency of tumor cells on activated signaling pathways driven by these genes.^298^, ^299^ For example, in NSCLC patients with mutations in driver genes such as *EGFR, ALK, and ROS1*, after resistance to first‐ and second‐generation TKIs, even upon receiving third‐ or fourth‐generation drugs, the targeting of driver gene signals persists, and some patients still respond effectively.^300^, ^301^ Additionally, some patients who develop resistance may still benefit from TKI rechallenge,^252^, ^302^ confirming the continued dependency of tumor cells on driver gene signals even after the development of resistance. This so called “driver gene addiction” persists after the development of TKI acquired resistance, leading many clinicians to continue TKI with subsequent chemotherapy.^303^ Current retrospective data demonstrated that continued first‐generation EGFR‐TKIs erlotinib and gefitinib with subsequent chemotherapy after progression was tolerated. Improved response rate and PFS were observed, especially in T790M‐negative patients.^303^, ^304^ However, results from the prospective IMPRESS trial did not demonstrate a clinical benefit of continuing gefitinib with chemotherapy after disease progression. Another two retrospective studies analyzed outcomes of osimertinib pretreated NSCLC patients receiving osimertinib plus chemotherapy as second‐line or later therapy. The median duration of treatment was 6.1 and 6.9 months in patients receiving platinum‐doublet chemotherapy and osimertinib, respectively.^305^, ^306^ Platinum‐based chemotherapy plus ALK‐TKI also shows modest efficacy in *ALK*‐positive NSCLC after failure of second‐generation ALK‐TKIs. A retrospective study demonstrated longer PFS in patients who received platinum/pemetrexed in combination with an ALK‐TKI compared with those who received platinum/pemetrexed alone (6.8 vs. 3.2 months, respectively). Although the aforementioned studies extended PFS compared with chemotherapy alone, the overall benefit remains limited, and there is a lack of large‐sample prospective clinical studies. Further prospective clinical trials are needed to investigate the potential benefit of TKI‐chemotherapy combination therapy after resistance.

Combining antiangiogenic inhibitors with chemotherapy is a more widely used combination regimen in clinical practice. Antiangiogenic inhibitors can normalize the surviving tumor vasculature, facilitating the delivery of chemotherapy drugs into the tumor tissue, and thereby enhancing the efficacy of chemotherapy.^307^, ^308^ Multiple studies have confirmed the efficacy of combining antiangiogenic drugs with conventional chemotherapy in various fields such as breast cancer, colorectal cancer, and NSCLC.^274^, ^308^, ^309^, ^310^ For NSCLC patients who develop resistance to targeted therapies like EGFR and ALK inhibitors, the current standard treatment recommended by guidelines is chemotherapy combined with bevacizumab.^311^

#### 3.2.4 Immunotherapy‐based combinations

Combining TKIs with ICIs has shown promising results in certain cancers, especially renal cell carcinoma and HCC. Currently, five of the six approved targeted therapy and immunotherapy combinations target angiogenesis. The VEGF–VEGFR pathway plays a key role in almost all immune cell subsets. VEGFRs are expressed on activated and memory T cells.^312^ VEGF–VEGFR is involved in the activation of downstream signaling pathways of T cells and inhibits the cytotoxic activity of T cells.^313^, ^314^ Antiangiogenesis agents, such as levantinib, axitinib, bevacizumab, and cabozantinib, have been reported to improve CD8+ T cell infiltration and activation via tumor vasculature normalization and suppression of inhibitory immune checkpoints including programmed death‐1 (PD‐1) and cytotoxic T‐lymphocyte‐associated protein 4 (CTLA4).^315^, ^316^ Consequently, multiple studies are now investigating the combination strategy of VEGFR inhibitors with PD‐1 or PD‐L1 ICIs to improve the efficacy. In 2021, the US FDA approved the combination of multikinase inhibitor lenvatinib plus PD‐1 antibody pembrolizumab for first‐line treatment of advanced renal cell carcinoma. The efficacy of this combination was investigated in CLEAR (NCT02811861), a multicenter, open‐label, randomized phase III trial in patients with advanced RCC in the first‐line setting. Patients receiving the combination regimen had a median PFS of 23.9 months compared with 9.2 months for those receiving sunitinib. The objective response rates were 71 and 36%; complete response rates were 16 and 4% on the combination and sunitinib arms, respectively.^317^ Inspiringly, the combination of pembrolizumab or avelumab (anti‐PD‐L1 antibody) with another small molecule antiangiogenesis agent axitinib has been approved for the treatment of patients with advanced RCC due to the encouraging results of KEYNOTE 426^318^ and JAVELIN Renal 101 trials.^319^

In addition to antiangiogenesis, some kinase inhibitors can potentially stimulate immune responses by preventing the increased expression of PD‐L1, enhancing T cell infiltration or inducing immune cell death.^320^, ^321^ For example, BRAF inhibition enhances melanoma antigen expression and facilitates T‐cell cytotoxicity, leading to a more favorable TME. This provides support for the potential synergy of BRAF‐targeted therapy and immunotherapy.^321^ The combination of BRAF inhibitor vemurafenib and the MEK inhibitor cobinetinib, along with atezolizumab, has now been approved for *BRAF*‐V600‐mutant melanoma.^322^ However, whether used in monotherapy or combination with chemotherapy, ICIs appear to be ineffective in *EGFR, ALK, RET*, *and HER2* driver gene‐positive cancers.^323^, ^324^, ^325^, ^326^, ^327^ This may be attributed to the small number of CD8+ tumor‐infiltrating lymphocytes and the low tumor mutational burden of these tumors, characteristics associated with a “cold” immune environment.^323^, ^328^, ^329^, ^330^ IMpower150 trial was the only positive clinical data on the efficacy of immunotherapy for both EGFR‐mutated and ALK‐rearranged tumors, which combined four drugs including atezolizumab, platin‐based chemotherapy, and bevacizumab. Only the combination of atezolizumab and bevacizumab with chemotherapy could provide benefit in EGFR‐TKI‐mutant patients.^331^ However, only one patient in the quadruplet therapy group had previously received osimertinib. Thus, it is impossible to draw a conclusive statement regarding the efficacy of the quadruplet therapy in the specific context of EGFR‐TKI failure. Moreover, an increased risk of severe toxicities with concomitant or sequential use of ICIs and specific EGFR or ALK‐TKIs was demonstrated, particularly grade 3−4 pneumonitis and hepatitis.^332^, ^333^, ^334^ Another phase II clinical study of ILLUMINATE explored the efficacy and safety of dual immunotherapy (anti‐PD‐L1 durvalumab and anti‐CTLA4 tremelimumab) combined with chemotherapy in metastatic EGFR‐mutation‐positive NSCLC that has progressed after EGFR‐TKI resistance (NCT03994393). The results of the study found that the dual immunotherapy combined chemotherapy brings moderate antitumor activity, with potential increased benefits in patients with EGFR T790M negative and PD‐L1 > 50%. The incidence of grade 3−4 immune‐related adverse events was 17%, mainly colitis and hepatitis.^335^ Overall, the available data do not support the general use of ICIs among patients with driver gene mutations following progression on targeted therapies. US FDA‐approved combination therapy of immunotherapy and TKI is summarized in Table 7.

**TABLE 7:** US FDA‐approved combination therapy of immunotherapy and tyrosine kinase inhibitor.

Indication	TKI	Immunotherapy	Year of approval	Trial name and references
First‐line advanced RCC	Axitinib	Pembrolizumab	2019	KEYNOTE‐426^318^
First‐line advanced RCC	Axitinib	Avelumab	2019	JAVELIN Renal 101^319^
First‐line advanced RCC	Lenvatinib	Pembrolizumab	2021	CLEAR^317^
First‐line advanced RCC	Cabozantinib	Nivolumab	2021	CheckMate 9ER^336^
First‐line unresectable HCC	Bevacizumab	Atezolizumab	2020	IMbrave 150^337^
First‐line BRAF V600E advanced melanoma	Vemorafenib+cobimetinib	Atezolizumab	2020	IMspire 150^322^
Endometril cancer (not MSI‐H or dMMR)	Lenvatinib	Pembrolizumab	2019	KEYNOTE‐146^338^

Abbreviations: dMMR, mismatch repair deficient; HCC, hepatocellular carcinoma; MSI‐H, microsatellite instability‐high; RCC, renal cell carcinoma; TKI, tyrosine kinase inhibitor.

### 3.3 Novel dual‐targeted bispecific antibodies or antibody–drug conjugates

Antibody–drug conjugates (ADCs) and dual‐targeted bispecific antibodies represent innovative therapeutic approaches that are currently in development and hold promise for patients with TKI‐resistant cancers. These novel agents are especially intriguing due to their ability to potentially address a wide range of resistance mechanisms. Amivantamab is a bispecific antibody targeting *EGFR* and *MET* that received accelerated approval for patients with NSCLCs harboring *EGFR* exon 20 insertion mutations in May 2021 based on the results of the CHRYSALIS trial.^339^ The combination of amivantamab with a third‐generation EGFR‐TKI lazertinib in patients with osimertinib‐relapsed, chemotherapy‐naïve, *EGFR*‐mutated advanced NSCLC has shown preliminary efficacy, with an ORR of 36% and a median PFS of 4.9 months.^264^ Inspiringly, amivantamab plus carboplatin–pemetrexed chemotherapy with and without lazertinib also demonstrated antitumor activity in patients with osimertinib refractory *EGFR*‐mutated advanced NSCLC. Objective response rate was significantly higher for amivantamab–chemotherapy and amivantamab–lazertinib–chemotherapy versus chemotherapy (64 and 63 vs. 36%, respectively).^263^ Of note, an exploratory analysis suggested that EGFR/MET immunohistochemistry (IHC) staining may predict for response to amivantamab plus lazertinib. A retrained signature of MET 3+ stating on 25% tumor cells was identified as predictive of response regardless of molecular resistance mechanism.^340^, ^341^

As another example, patritumab deruxtecan is a specifically engineered potential first‐in‐class HER3‐directed ADC. In HERTHENA‐Lung01, patritumab deruxtecan was studied in 225 patients with EGFR‐mutated locally advanced or metastatic NSCLC following disease progression with an EGFR‐TKI and platinum‐based chemotherapy, which demonstrated an ORR of 29.8%. The median duration of response was 6.4 months.^342^ An analysis of 48 paired pretreatment and posttreatment tumor samples showed augmented HER3 expression in *EGFR*‐mutant tumors with acquired resistance to EGFR‐TKIs^343^ and that EGFR inhibition increased membrane expression of HER3 and led to enhanced efficacy of HER3‐DXd,^344^ thus supporting the rational combination of osimertinib and HER3‐DXd in *EGFR*‐mutant NSCLC. The activity of patritumab deruxtecan in patients with previously treated *EGFR*‐mutant NSCLCs is currently being investigated further, both as a monotherapy (NCT04619004) and in combination with osimertinib (NCT04676477). BL‐B01D1, a bispecific topoisomerase inhibitor‐based ADC that targets both EGFR and HER3, is currently being evaluated in a phase I study (BL‐B01D1‐LUNG101; NCT05194982) for safety and efficacy in individuals with metastatic or unresectable NSCLC. Data from earlier clinical studies of BL‐B01D1 were presented in 2023 at the American Society of Clinical Oncology, European Society for Medical Oncology (ESMO) and the San Antonio Breast Cancer Symposium; these data demonstrated impressive antitumor activity in patients with EGFR‐TKI resistance, with an ORR of 63.2%.^345^ Both EGFR and HER3 are highly expressed in most epithelial tumors; thus, BL‐B01D1 may have promising antitumor activity in patients with a range of solid tumors.

Trastuzumab deruxtecan (DS‐8201, known as dequdacimab), a HER2‐targeted ADC, has shown significant clinical efficacy in patients with lung cancer who harbor *HER2* gene alterations. The treatment of advanced *HER2*‐mutant NSCLC has historically been guided by the approach for advanced NSCLC without driver gene mutations. HER2‐TKIs and anti‐HER2 antibodies have shown limited efficacy in this setting,^346^, ^347^ while ADC drug have achieved breakthrough progress in *HER2*‐mutant NSCLC. The DESTINY‐Lung01 and DESTINY‐Lung02 studies have demonstrated the efficacy of DS8201 in HER2‐mutant NSCLC who have progressed after standard treatment or targeted HER2‐TKI therapy, with ORR of 54.9 and 53.8%, respectively, and a median duration of response of 8.2 months.^348^, ^349^ In the DESTINY‐PanTumor02 trial, which included 267 patients covering various tumor types such as endometrial cancer, cervical cancer, ovarian cancer, bladder cancer, and bile duct cancer, DS‐8201 demonstrated significant efficacy in multiple HER2‐positive solid tumors. In IHC3+ HER2‐positive patients, the ORR reached an impressive 61.3%, with a PFS of 11.9 months and a median duration of response (DOR) of 22.1 months.^350^ Based on this study, on April 5, 2024, the US FDA granted accelerated approval to trastuzumab deruxtecan for previously treated and no further treatment options available, unresectable or metastatic HER2‐positive (IHC3+) solid tumors. This marks a significant breakthrough for DS8201, allowing for potential treatment across multiple cancer types.

The potential role of alternative ADCs targeting the human trophoblast cell‐surface antigen 2 (TROP2) (NCT04152499) and MET^351^, ^352^ (NCT03859752) is also being evaluated in patients with lung cancers, potentially including NSCLCs with *EGFR* mutations or *ALK* rearrangements. TROP2 is highly expressed in various solid tumors and is associated with worse prognosis.^353^ It has emerged as a hot target in the field of oncology in recent years, and the development of ADCs targeting this antigen has garnered significant attention. SKB264 (MK‐2870) is a novel anti‐TROP2 ADC. In a phase II study, 22 cases of EGFR mutation patients were enrolled who experienced progression during or after TKI treatment, 50% of patients had experienced at least one chemotherapy failure. The ORR was 60%, with a median PFS of 11.1 months.^354^ The ongoing phase III MK‐2870‐004 study (NCT06074588) will evaluate the efficacy and safety of MK‐2870 versus chemotherapy for previously TKI‐treated NSCLC with *EGFR* mutations or other genomic alterations in *ALK, ROS1, BRAF V600E, NTRK, MET* exon 14 skipping, or *RET* and those with less common *EGFR* mutations (exon 20 S768I, exon 21 L861Q, and/or exon 18 G719X). ABBV‐399 (also called Telisotuzumab‐Vedotin) is currently the only MET‐targeting ADC that has entered phase III clinical trials. In January 2022, ABBV‐399 was granted breakthrough therapy designation by the US FDA for the treatment of advanced or metastatic MET overexpressing EGFR WT nonsquamous NSCLC patients who have progressed after platinum‐based therapy. In the single‐arm phase II LUMINOSITY trial (NCT03539536), ABBV‐399 demonstrated promising results in the treatment of MET protein overexpressing, EGFR WT, advanced/metastatic non‐squamous NSCLC. The ORR was 53.8% in the high MET expression group and 25.0% in the moderate MET expression group, with a median duration of response of 9 and 7.2 months, respectively.^355^ ABBV‐399 is not the only MET ADC drug in development, others like MYTX‐011(NCT05652868), SHR‐A1403(NCT03856541), TR1801(NCT03859752), and more are also in clinical trial stages. Targeting MET with ADCs provides a new treatment option for patients who develop MET overexpression after developing resistance to previous therapies.

### 3.4 PROteolysis targeting chimeras

PROteolysis targeting chimeras (PROTAC) is a molecular tool designed for targeted protein degradation. It is a heterobifunctional compound typically composed of three main parts: a binding moiety to target the protein of interest, a binding moiety to recruit the cellular ubiquitin E3 ligase, and a linker.^356^, ^357^ The mechanism of PROTAC relies on the dual binding of the target protein and the E3 ligase. When PROTAC binds to both the target protein and the E3 ligase, it forms a ternary complex. The E3 ligase then ubiquitinates the target protein, attaching a chain of ubiquitin molecules to it. The ubiquitinated target protein is recognized and degraded by the cell's ubiquitin‐proteasome system, ultimately leading to the degradation of the target protein.^358^, ^359^ Traditional small molecule drugs may require higher drug concentrations to effectively inhibit the activity of the target protein, whereas PROTAC molecules can be reused after the degradation of ubiquitinated proteins, resulting in lower effective drug concentrations needed for PROTAC action.^360^, ^361^ Additionally, compared with traditional reversible inhibition, targeted degradation provides a longer duration of continuous blockade of the target protein, as the degraded protein needs to be resynthesized.^360^, ^361^ These advantages make PROTAC particularly suitable for addressing resistance caused by acquired mutations or amplifications in cancer.

Targeting proteins for proteasomal degradation using the PROTAC approach is now widely pursued in clinical development, mainly targeting proteins in cancer (e.g., BCR–ABL,^362^, ^363^ EGFR,^361^, ^364^, ^365^ androgen receptor (AR),^366^, ^367^, ^368^ estrogen receptor (ER),^369^, ^370^ bromodomain‐containing protein 4 (BRD4),^371^ or Bruton's kinase (BTK)^372^, ^373^). Bavdegalutamide (ARV‐110) is an orally active, specific AR PROTAC degrader, which is in development for the treatment of men with metastatic castration‐resistant prostate cancer (mCRPC) who have progressed on abiraterone and/or enzalutamide.^374^ Phase I/II study of ARV‐110 (NCT0388861) demonstrated clinical activity in heavily pretreated mCRPC, with the greatest PSA50 activity and RECIST responses in patients with AR T878 and/or H875 mutations.^368^ Another example that demonstrates the clinical impact of this strategy is the BTK degrader developed to tackle on‐target resistance to BTK inhibitors. The most common resistance mechanism in patients whose disease progresses on covalent BTK inhibitors is a mutation in the BTK C481 residue, abrogating the covalent binding of irreversible BTK inhibitors such as ibrutinib.^72^, ^91^ PROTAC technology itself does not require a strong affinity between the binder and the target protein. In fact, it has been shown that PROTAC molecules with ibrutinib as the binder, although having weaker binding to BTK C481S, can induce degradation of the BTK protein.^375^ This indicates that protein kinase conferring resistance to anticancer drugs may be targeted using the PROTAC approach.

## 4 FUTURE DIRECTIONS IN RESEARCH AND CLINICAL PRACTICE

The discovery of actionable driver oncogenes and TKIs launched a paradigm shift in cancer treatment. As TKI‐targeted therapy continues to evolve, future directions emphasize several pivotal areas of research and development aimed at overcoming resistance and improving treatment efficacy. Here, we focus on discussing future development strategies in TKI‐targeted therapy, including the monitoring of resistance through liquid biopsies, the development of novel kinase inhibitors, and the new nanoscale delivery systems.

### 4.1 Methods for detecting and monitoring resistance during treatment

Early monitoring of drug resistance mutations can determine the best treatment regimen, particularly with the development of high‐quality detection methods in recent years. The current approach to disease management revolves around tumor‐specific, stage‐based guidelines, primarily relying on pathology and imaging outcomes to optimize patient treatment plans.^376^ Imaging techniques such as CT scans, MRI, or PET scans are valuable for monitoring disease progression or relapse and assessing treatment response; however, interpreting radiological findings can be challenging, resulting in high rates of false positives and negatives.^377^ Serial monitoring of tumor markers, such as carcinoembryonic antigen, cancer antigen (CA)‐125, CA19‐9, PSA, and lactate dehydrogenase, offers a noninvasive means to assess treatment response. However, many of these established biomarkers are deemed unreliable due to potential elevation from noncancer‐related conditions, leading to reduced sensitivity and specificity.^378^, ^379^, ^380^ NGS allows for the simultaneous analysis of multiple genes and molecular alterations in tumor samples. This provides a comprehensive view of the genomic landscape of cancer, including driver mutations, copy number alterations, and gene fusions.^381^ Currently, NGS testing is based on tissue biopsy at the time of diagnosis and, when possible, a rebiopsy at the time of progression remains the gold standard, providing direct analysis of genetic mutations or alterations indicative of resistance.^382^ However, tissue samples are not always feasible, and the sample amount is often insufficient for molecular detection.^383^ Additionally, tissue samples are not often enough to systematically represent the mutational status of the entire tumor due to tumor heterogeneity and clonal dynamics. In contrast, liquid biopsy offers a minimally invasive alternative, analyzing circulating tumor cells or cell‐free DNA to detect real‐time genetic changes associated with resistance.^384^ Dynamic sequencing analysis of circulating tumor DNA (ctDNA) has multiple potential clinical applications, including screening, distinguishing early‐stage diseases, detecting molecular residual disease after curative surgery, predicting recurrence, genotyping advanced tumors, early assessment of treatment efficacy, monitoring treatment response, and identifying mechanisms of resistance, serving as an effective alternative to tissue‐based genetic testing.^385^, ^386^, ^387^, ^388^ For example, in the monitoring of advanced colorectal cancer, ctDNA enables the dynamic and repeated assessment of changes in multiple key molecules of the tumor, such as EGFR, ERBB2, PIK3CA, and MAP2K1, among others. This facilitates the identification of novel potential therapeutic targets and mechanisms of resistance to targeted therapies, including anti‐EGFR, anti‐BRAF, and anti‐HER2 drugs. Studies have demonstrated a high concordance between ctDNA detection results and tissue biopsy outcomes.^386^, ^387^, ^388^ Moreover, ctDNA is advantageous due to its ability to capture molecular heterogeneity within and between tumors, unrestricted by sampling location.^389^, ^390^ Dynamic sequencing of ctDNA was also able to decode the evolutionary response of NSCLC patients who received targeted therapy. This approach effectively guides further research directions and clinical treatment practices.^391^, ^392^ In metastatic breast cancer, ctDNA demonstrates higher accuracy compared with standard serum markers like CA15‐3. Dynamic changes in ctDNA are associated with PFS during chemotherapy, endocrine therapy, and combination targeted therapy.^393^, ^394^, ^395^, ^396^ Additionally, sequential ctDNA analysis can also be employed to assess genomic mechanisms of resistance that arise before clinical progression. In colorectal cancer patients receiving anti‐EGFR monoclonal antibody therapy, RAS and EGFR‐extracellular domain mutations were detected in ctDNA 10 months before radiographic progression, and these mutations disappeared after discontinuation of anti‐EGFR therapy.^397^, ^398^ For advanced breast cancer patients receiving aromatase inhibitor (AI) and CDK4/6 inhibitor therapy, monitoring the emergence of estrogen receptor 1 (*ESR1*) mutations holds potential clinical utility. In the PADA‐1 trial, patients with detected *ESR1* mutations were randomized to receive fulvestrant with continued CDK4/6 inhibitor therapy, when this group was compared with those continuing AI and CDK4/6 inhibitor therapy, an improvement in PFS was observed. The PFS benefit observed with early fulvestrant use after detection of *ESR1* mutations may not be compensated for by later fulvestrant use after disease progression.^399^ These data suggest that detecting resistance‐related mutations in ctDNA earlier rather than later (at radiographic progression) may have more decision‐making significance. Although ctDNA holds immense potential as a minimally invasive biomarker for tumor monitoring, its clinical application still faces several limitations and challenges. The optimal timing for dynamic assessment of ctDNA and the most accurate efficacy prediction thresholds require further investigation. To monitor the emergence of resistance mutations before clinical progression, further research is needed to determine the frequency of monitoring. Randomized intervention studies are necessary to assess whether changing treatment based on dynamic ctDNA assessment can improve prognosis or even replace current standard clinical metrics.

### 4.2 Identification of novel targets

The future of kinase inhibitors partly depends on the identification of novel targets. Current targeted kinase inhibitor therapies focus mostly on known tyrosine kinases, yet there are upward of 500 total kinases in the human genome. During the past 5 years, the number of small molecule kinase inhibitors entering clinical development has increased by over 200%.^229^ Currently, investigational kinase inhibitors in clinical trials target approximately 110 new kinase targets, accounting for 20% of the human kinome,^229^ reflecting the active exploration of innovative targets in the kinase field.

Aurora kinase family (Aurora kinase A, Aurora kinase B and Aurora kinase C) has been the most targeted kinase family in oncology, with over 20 drugs entering clinical trials. Aurora kinases are crucial mitotic regulators required for genome stability, and they are often overexpressed in human cancers such as breast cancer, ovarian cancer, gastrointestinal cancer, and this abnormal expression is associated with poor prognosis in cancer patients.^400^ In recent years, increasing evidence suggests that Aurora inhibitors hold promise for cancer therapy and overcoming resistance to inhibitors targeting CDK4/6 and EGFR.^401^, ^402^ Even though Aurora inhibitors hold treatment potential in oncology, so far, no Aurora kinase inhibitor has been approved for clinical use because of the limited clinical efficacy and drug toxicity. Currently, second‐generation, isoform‐selective Aurora kinase inhibitors are being investigated as monotherapies or in combinations (etc., LY3295668 combined with osimertinib (NCT05017025), MLN8237 and bortezomib in treating multiple myeloma (NCT010345535), VIC‐1911 combined with lenvatinib (NCT05718882)) for hematological malignancies and solid tumors.

The CDK family has been a therapeutic target for over 20 years because of its critical roles in controlling cell division, and cancers often show dysregulation of the cell cycle.^403^ From 2015 to 2017, selective inhibitors targeting CDK4 and CDK6 gained US FDA approval for treating breast cancer, including palbociclib, abemaciclib, and ribociclib. Members of the CDK family continue to be a hotspot for the development of small‐molecule kinase inhibitors. Apart from CDK4 and CDK6, there are currently multiple small molecule inhibitors targeting CDK7, CDK8/19, CDK2, and CDK9. CDK2, a critical regulator of the cell cycle, is a promising drug target, especially in cancers with cyclin E1 protein overexpression, including HR+/HER2− breast cancer resistant to CDK4/6 inhibitors.^404^ Multiple companies, including AstraZeneca, Pfizer, and Blueprint Medicines, are actively developing CDK2 inhibitors. Currently, several CDK2 inhibitors have entered clinical trial stages, being tested both as monotherapy and in combination with CDK4/6 inhibitors.

Hematopoietic progenitor kinase 1 (HPK1) is a family member of mitogen‐activated protein kinase kinase kinase kinase, which is a kinases that catalyze the phosphorylation of serine or threonine residues in proteins.^405^, ^406^ HPK1 is an intracellular negative regulator of T/B‐cell proliferation and signaling. HPK1 kinase knockdown mice demonstrate increased CD8+ T‐cell killing function and robust antitumor immune responses even in a cold tumor environment, making HPK1 a high‐priority target in immuno‐oncology.^407^, ^408^ Several lines of preclinical evidence have been collected over the past years that support the development of compounds targeting HPK1, especially in combination with anti‐PD‐1/PD‐L1 ICIs. In 2020, Treadwell Therapeutics' HPK1 inhibitor CFI‐402411 became the first to enter phase I clinical trials (NCT04521413). Subsequently, orally highly selective HPK1 inhibitors such as Pfizer's PF‐07265028 (NCT05233436), BeiGene's BGB‐15025 (NCT04649385), and Zhuhai Yufan Biotechnologies’ PRJ1‐3024 (NCT05315167) have entered phase I/II clinical trials. These inhibitors are primarily being investigated as monotherapy or in combination with PD‐1/PD‐L1 inhibitors for the treatment of advanced solid tumors.

SHP2 is a phosphatase that dephosphorylates substrate proteins. It is a “star target” in the field of cancer therapy. In the RTK pathway, SHP2 effects are upstream of RAS, in mediating tumor proliferation. Virtually all RTKs initiate the RAS signaling pathway by activating SHP2. Thus, inhibitors of SHP2 can block the RTK/RAS/MAPK signaling pathways and inhibit the growth and proliferation of tumor cells driven by RTK or with KRAS, BRAF Class 3 and NF1 loss of function mutations.^245^, ^246^, ^247^, ^248^, ^409^ SHP2 effects are also downstream of the immune checkpoint regulator, PD‐1, in inhibiting the antitumor effect of T cells.^410^, ^411^, ^412^ Given the clinical success of anti‐PD‐1/PD‐L1 immunotherapy, SHP2 inhibitors serve as small molecule tumor immunotherapeutic agents and are considered important complements to PD‐1/PD‐L1 inhibitors, holding great promise in clinical practice. SHP2 also functions downstream of colony stimulating factor 1 receptor, in promoting TAM activity. Thus, targeting SHP2 has the potential for multiple antitumor effects. Currently, there are no drugs targeting the SHP2 target approved for market, but several SHP2 inhibitors have entered clinical trials. These include Novartis’ TNO155 (NCT03114319), Sanofi's RMC‐4630 (NCT04916236), Etern BioPharma's ET0038 (NCT05354843) and Jacobio Pharma's JAB‐3312 (NCT04720976). The main indications for these inhibitors include combination therapy with KRAS G12C inhibitors, EGFR inhibitors, BRAF inhibitors, MEK inhibitors, ERK inhibitors, and anti‐PD‐1/PD‐L1 antibodies. Among them, the most rapidly advancing is JAB‐3312, which has become the world's first SHP2 inhibitor to be approved for phase III pivotal clinical trials in combination with KRAS G12C inhibitors, leading the field of SHP2 inhibitors. Data presented at the 2023 ESMO Congress revealed that in the dose group combining the KRAS G12C inhibitor sotorasib with the SHP2 inhibitor JAB‐3312, the objective response rate was 50%, with a disease control rate of 100%.^413^

At least 70% of the kinome is still unexplored in drug development. New large‐scale screening methods such as large‐scale quantitative proteomic screens and scRNA‐seq may help identify transcript levels of kinases that drive clonal evolution or transcriptional reprogramming, holding promise for the development of novel targeted kinase inhibitor therapies.

### 4.3 Dual‐acting kinase inhibitors

As mentioned above, combination therapy theoretically may have additive or synergistic effects; however, it often leads to unpredictable side effects, such as increased toxicity. As an alternative strategy to combination therapy, dual‐target or multitarget drugs are considered to have lower risks of drug–drug interactions and better pharmacokinetics (PK) and safety profiles. Compared with combination therapy, these drugs mitigate issues such as reduced bioavailability, metabolism, and antagonistic effects. The advantage of this approach lies in its ability to simultaneously target multiple points, thereby providing a more comprehensive therapeutic effect against complex disease biology processes. By reducing interactions between drugs, dual‐target or multitarget drugs may help reduce the risk of side effects and improve the safety and tolerability of treatment. Currently, many dual‐target drugs have been investigated in cancer treatment. For example, alectinib is a dual‐target small molecule inhibitor that simultaneously targets ALK and RET. Next‐generation RET‐TKI TPX‐0047 simultaneously targets RET and SRC.^414^ The dual‐target CHK1/FLT3 inhibitor TLX83 can overcome adaptive and acquired resistance to FLT3 inhibitors, offering a new strategy for patients with *FLT3‐ITD*‐mutant acute myeloid leukemia (AML).^415^ The development of EGFR dual‐target inhibitors is a promising strategy to decrease the potential drug resistance of EGFR‐TKIs induced by aberrant alternative signaling pathways. Preclinical studies have shown that the combination of erlotinib and HDAC inhibitors promotes apoptosis in TKI‐resistant NSCLC cells.^416^ CUDC‐101, a multitarget HDAC/EGFR/HER2 inhibitor, has shown effective inhibitory activity against advanced solid tumors in vivo.^417^ Phase I study (NCT00728793) demonstrated CUDC‐101 was generally well tolerated with preliminary evidence of antitumor activity.^418^ A new MEK inhibitor currently in clinical trials for metastatic melanoma (NCT04720417) simultaneously inhibits RAF feedback activity. So far, various design strategies for dual‐target inhibitors targeting different tumor types and pathways have been reported.

As mentioned above, the future development of kinase inhibitors faces challenges beyond the discovery of new targets, particularly in developing compounds with high clinical efficacy and low risk of off‐target toxic side effects and resistance. In this regard, developing inhibitors with different binding modes, such as allosteric and covalent inhibitors, as well as developing dual‐acting kinase inhibitors, or targeted protein degraders, may play a crucial role in kinase drug development in the next 20 years.

### 4.4 Nanoparticle‐based drug delivery systems

The combination of nanotechnology and targeted therapy has emerged as a promising field in cancer research. Nanoparticle‐based drug delivery systems, including polymeric, lipid, carbon nanotubes, dendrimer, and micellar nanoparticles have been extensively designed and investigated in preclinical cancer treatment.^419^, ^420^, ^421^ Compared with the conventional delivery routes, nanoparticle‐based drug delivery systems possess some unique advantages with improved PKs, increased efficacy, and reduced off‐target cytotoxicity. For example, nanoparticle‐based drug delivery systems can be utilized to codeliver two different drugs or reagents concurrently. A codelivery system of afatinib and paclitaxel was designed for the treatment of EGFR‐TKI‐resistant NSCLC.^422^ The results of this study showed that afatinib and paclitaxel from the system can sustain high concentrations in the lung for up to 96 h, while maintaining lower concentrations in other tissues in Sprague–Dawley rats.^422^ Besides, as mentioned above, hypoxia in solid tumors can mediate acquired resistance to TKI therapy. Li et al.^423^ codelivered oxygen‐releasing perfluorooctylbromide with erlotinib in liposomal nanoparticles to address hypoxia‐induced drug resistance. They found the codelivery system could significantly improve the efficacy of erlotinib when compared with nanoparticles loaded with erlotinib alone.^423^

However, nearly all US FDA‐approved nanomedicines are formulated for intravenous (i.v.) use, while the majority of TKIs are designed for oral. More advanced nanocarriers capable of overcoming the challenges of oral administration will be needed. In addition, most of these studies are limited to preclinical settings. Nano‐applications of TKIs are still in their infancy stages of development, and yet to be validated in clinical practice. Despite their immense potential, they still have a considerable journey ahead of them.

### 4.5 Targeting drug‐tolerant persistent cells (DTP cells)

In recent years, DTP cells have gained considerable attention as a promising area in cancer research, particularly regarding their role as nongenetic mechanisms of therapeutic resistance.^424^ DTP cells are a subpopulation of cancer cells that survive exposure to targeted therapy, despite the treatment being initially effective against the bulk of tumor cells.^425^ These cells can enter a quiescent or slow‐proliferative state, allowing them to evade the cytotoxic effects of the drug.^425^, ^426^ While DTP cells may initially respond to treatment, they can eventually lead to disease recurrence or the development of drug resistance.^425^, ^426^ Understanding and targeting DTP cells is crucial for improving the long‐term efficacy of cancer‐targeted therapies.

In cancer, DTP cells were first found and described in the *EGFR*‐mutant NSCLC cell line PC9 by Sharma and colleagues in 2010.^427^ They observed that these cells exhibited over 100‐fold lower drug sensitivity and could revert to their original state upon drug withdrawal.^427^ An epigenetic regulator, histone demethylase KDM5A, was found to be required for the establishment of DTP cells, and histone deacetylase (HDAC) inhibitors could ablate the generation of DTP cells.^427^ Subsequently, DTP cells have been identified in various experimental models after targeted therapy, including additional *EGFR*‐mutant NSCLC cell lines, *MET*‐amplified gastric cancer, *BRAF*‐mutant melanoma, prostate cancer, colorectal cancer, and *HER2*‐positive breast cancer.^428^, ^429^, ^430^, ^431^, ^432^, ^433^ Two common phenotypic features of DTP cells, including slow proliferation and reversible biological capacity, have been well documented in different studies.^434^ However, the mechanisms responsible for regulating DTP state are context‐specific across various cancer models and therapeutic approaches, which include diverse epigenetic reprogramming, transcriptional regulation, and metabolic remodeling.^425^, ^434^

Despite accumulating evidence regarding the existence of DTP cells and the putative mechanisms underlying the regulation of DTP state, universally accepted biomarkers for DTP cells remain elusive. The definition of cancer persistent cell state is yet to be established. Besides, most studies characterized DTP cells by using in vitro cell line experimental models,^427^, ^428^ with few studies employing in vivo patient‐derived xenograft mouse models.^431^, ^435^ Data on patients are still limited. Our clinical understanding of their roles in cancer treatment resistance remains unclear, and therapeutic strategies targeting DTP cells have yet to be explored. Based on the concept of DTP cells, a potential therapeutic strategy of withdrawing targeted therapy and enabling tumor repopulation with drug‐sensitive cells has been discussed. However, tumor evolution is unpredictable. What's more, clinical experience over decades has demonstrated attempting to reintroduce the same therapy to a patient is consistently futile.^424^ Additionally, DTP cells have been proven to be highly heterogeneous and context‐specific. With the advancement of single‐cell multiomics technologies, including spatial transcriptomics, epigenomics, proteomics, and metabolomics, a more comprehensive understanding of DTP cells may be established and elucidated.

### 4.6 Investigating resistance mechanisms at single‐cell resolution

Most resistance mechanisms discussed above were uncovered through the analysis of bulk tumor specimens. Considering that tumors are highly heterogeneous, bulk‐tumor analysis may not completely reveal the entire spectrum of resistance mechanisms.^436^ Single‐cell analyses of matched tumor specimens or cell line models before and after treatment are informative in identifying potential mechanisms of TKI‐therapy resistance in various studies.^432^, ^437^, ^438^, ^439^, ^440^ For example, by performing single‐cell RNA sequencing (scRNA‐seq) and single‐cell ATAC‐sequencing analysis on both TKI‐resistant cells and clinical specimens, Kashima and colleagues identified CD74 as a novel key factor in regulating tumor drug‐tolerant state.^437^ Although single‐cell RNA‐seq holds promise in deciphering the transcriptomic profiles of tumor subclones, it lacks coverage of critical mutation information in cells. As we discussed above, most well‐reported resistance mechanisms are based on secondary mutations. An innovative genomic technology for single‐cell mutational analysis and concurrent transcriptomic sequencing will allow for further understanding of molecular mechanisms underlying treatment resistance, by providing insights into dysregulated pathways in both mutant and nonmutant tumor cells. Alba and colleagues developed such a method for the detection of hotspot mutations, in parallel with unbiased RNA‐seq analysis within single cells.^441^ However, this method has not yet been applied to comprehensively explore resistance mechanisms of cancer treatment.

Multiomics techniques, particularly NGS and scRNA‐seq, have revolutionized our understanding of tumor biology by uncovering new biomarkers and molecular regulators linked to oncogenesis, metastasis, and drug resistance.^438^, ^439^, ^440^, ^442^ Nonetheless, these methods have limitations as they do not capture the spatial organization of cells within the TME, thus providing an incomplete picture of tumor biology. There is an increasing demand for image‐based methodologies with single‐cell resolution to visualize intratumor heterogeneity, cell–cell interactions, and protein expression information within the TME. Emerging technologies that utilize multiplexed fluorescence, RNA, DNA, and isotope labeling now enable the deep investigation of cancer biology within their spatial context.^443^, ^444^ By conducting single‐cell multiomic assays including nucleus RNA, open‐chromatin, spatial transcriptomics, spatial proteomics and exome sequencing, on 86 matched primary‐recurrent tumor specimens, Lin and colleagues comprehensively described a single‐cell atlas of glioblastoma evolution under treatment.^445^

While multiomics techniques offer immense potential in cancer research, several challenges hinder their widespread application. One major obstacle is the resistance mechanisms identified by multiomics techniques are usually individual‐specific and context‐specific, making it difficult to develop universal therapeutic approaches applicable to all patients. Moreover, the cost and time required for multiomic analyses are substantial, limiting their accessibility and scalability. Besides, tumors exhibit high levels of heterogeneity, both within and between tumors. Thus, single‐cell resolution is crucial for capturing this heterogeneity, but it also introduces complexity to data analysis, further complicating the interpretation of results. Overcoming these challenges will require advancements in technology, computational methods, and collaborative efforts within the scientific community to fully leverage the potential of single‐cell multiomic technologies in cancer research.

## 5 CONCLUSIONS

TKI resistance poses significant clinical challenges, with multiple resistance mechanisms now elucidated. Recent progress in developing next‐generation TKIs, combination therapies, ADCs, and PROTACs has shown promise in overcoming these mechanisms, demonstrating encouraging results in clinical trials. However, many cancers treated with TKIs, such as NSCLC, exhibit high heterogeneity, leading to inevitable drug resistance. There is still an urgent need for novel therapeutic strategies either targeting TKI resistance or employing de novo strategies to extend patients’ lives and improve their life quality of life while living with cancer. The advancement of new technologies, including liquid biopsy, nanotechnologies, image‐based single‐cell methodologies, and multiomics approaches, holds potential for deeper insights into TKI resistance and tumor biology. These advancements can facilitate the development of innovative cancer treatment strategies.

## AUTHOR CONTRIBUTIONS

Xuejin Ou and Ge Gao wrote the manuscript. Inbar A. Habaz conducted a thorough review of relevant literature and provided critical revisions to the manuscript. Yongsheng Wang conceived the idea for the review paper, provided funding support, and edited the manuscript. All authors have read and approved the final manuscript.

## CONFLICT OF INTEREST STATEMENT

The authors declare that they have no known financial or nonfinancial interests that are directly or indirectly related to the work submitted for publication.

## ETHICS STATEMENT

Not applicable.

## Data Availability

Not applicable.
